# *CRSP8*-driven fatty acid metabolism reprogramming enhances hepatocellular carcinoma progression by inhibiting RAN-mediated PPARα nucleus-cytoplasm shuttling

**DOI:** 10.1186/s13046-025-03329-3

**Published:** 2025-03-11

**Authors:** Yuxi Lin, Zhixing Liang, Zhiyan Weng, Xiaofang Liu, Feng Zhang, Yutian Chong

**Affiliations:** 1 Department of Infectious Diseases, The Third Affiliated Hospital, Sun Yat-sen University, Guangzhou, 510630 China; 2 Guangdong Provincial Key Laboratory of Liver Disease Research, The Third Affiliated Hospital, Sun Yat-sen University, Guangzhou, 510630 China; 3 Department of Hepatic Surgery and Liver Transplantation Center, The Third Affiliated Hospital, Sun Yat-sen University, Guangzhou, 510630 China; 4 Department of Endocrinology, The First Affiliated Hospital, Fujian Medical University, Fuzhou, 350005 China; 5 Department of Neurology, The Third Affiliated Hospital, Sun Yat-sen University, Guangzhou, 510630 China; 6 Biotherapy Centre, The Third Affiliated Hospital, Sun Yat-sen University, Guangzhou, 510630 China

**Keywords:** CRSP8, Fatty acid oxidation, Lipophagy, Hepatocellular carcinoma, PPARα

## Abstract

**Background:**

In-depth exploration into the dysregulation of lipid metabolism in hepatocellular carcinoma (HCC) has contributed to the development of advanced antitumor strategies. *CRSP8* is a critical component of mediator multiprotein complex involved in transcriptional recruiting. However, the regulatory mechanisms of *CRSP8* on fatty acid metabolism reprogramming and HCC progression remain unclear.

**Methods:**

In-silico/house dataset analysis, lipid droplets (LDs) formation, HCC mouse models and targeted lipidomic analysis were performed to determine the function of CRSP8 on regulating lipid metabolism in HCC. The subcellular colocalization and live cell imaging of LDs, transmission electron microscopy, co-immunoprecipitation and luciferase reporter assay were employed to investigate their potential mechanism.

**Results:**

CRSP8 was identified as a highly expressed oncogene essential for the proliferation and aggressiveness of HCC in vitro and in vivo. The tumor promotion of *CRSP8* was accompanied by LDs accumulation and increased de novo fatty acids (FAs) synthesis. Moreover, CRSP8 diminished the colocalization between LC3 and LDs to impair lipophagy in a nuclear-localized PPARα-dependent manner, which decreased the mobilization of FAs from LDs degradation and hindered mitochondrial fatty acid oxidation. Mechanistically, the small *ras* family GTPase *RAN* was transcriptionally activated by *CRSP8*, leading to the reinforcement of RAN/CRM1-mediated nuclear export. CRSP8-induced enhanced formation of RAN/CRM1/PPARα nucleus-cytoplasm shuttling heterotrimer orchestrated cytoplasmic translocation of PPARα, attenuated nPPARα-mediated lipophagy and fatty acid catabolism, subsequently exacerbated HCC progression. In CRSP8-enriched HCC, lipid synthesis inhibitor Orlistat effectively reshaped the immunosuppressive tumor microenvironment (TME) and improved the efficacy of anti-PD-L1 therapy in vivo.

**Conclusion:**

Our study establishes that CRSP8-driven fatty acid metabolism reprogramming facilitates HCC progression via the RAN/CRM1/PPARα nucleus-cytoplasm shuttling heterotrimer and impaired lipophagy-derived catabolism. Targeting the energy supply sourced from lipids could represent a promising therapeutic strategy for treating CRSP8-sufficient HCC.

**Supplementary Information:**

The online version contains supplementary material available at 10.1186/s13046-025-03329-3.

## Introduction

Hepatocellular carcinoma (HCC) represents over 90% of all primary liver cancers and ranks as the second leading cause of cancer-related fatalities globally [1]. The poor prognosis of HCC can be attributed to its aggressive nature, elevated metastasis rates, treatment resistance, and recurrence propensity [2]. Consequently, it’s crucial to thoroughly investigate the mechanisms driving HCC progression, discover novel therapeutic approaches, and enhance current treatment options.

Metabolic reprogramming is a key feature of cancer [3]. Alongside established roles of glucose and glutamine metabolism, it’s acknowledged that lipid metabolism is also significant [4]. Many enzymes that play a role in fatty acid (FA) uptake, synthesis, lipolysis, and fatty acid oxidation (FAO) have been found to be irregularly regulated in various cancers. This dysregulation leads to the reprogramming of FA metabolism and supports malignant cancer characteristics, highlighting the importance of lipid metabolism disturbances [5]. Ongoing de novo lipogenesis provides cancers with a sufficient supply of lipids necessary for membrane formation and signal transduction, thereby facilitating cancer onset and advancement [6]. A disruption in the balance of intracellular lipid homeostasis, along with alterations in lipid and cholesterol levels, affects the tumor microenvironment (TME). Lipolysis has traditionally been understood to take place via the activity of cytosolic neutral lipase, which breaks down triglycerides (TGs) stored in lipid droplets (LDs) into free FAs and glycerol. Lipophagy was known as a specific type of autophagy, which breaks down lipids stored in LDs, emerged from the observation of lipolysis and macroautophagy [7]. Given the function of lipid metabolism in maintaining liver homeostasis, disrupted lipophagy may contribute to hepatic lipid metabolism reprogramming as HCC develops [8]. The degradation of lipids supplies energy to the cell via FAO and influences various cellular processes; for instance, it activates signaling pathways associated with carcinogenesis that promote the advancement of cancer [8]. It’s indicated that hepatic cancer cells utilize macroautophagy to facilitate tumor progression. Specifically, elevated macroautophagy markers like light chain 3 (LC3) were linked to unfavorable prognosis and increased recurrence rates following surgery [9]. Nonetheless, the involvement of LD metabolism, particularly lipophagy, in the advancement of HCC remains poorly understood. The molecular mechanisms connecting disrupted fatty acid metabolism to liver tumorigenesis are still unclear.

Identifying the key regulators of fatty acid metabolism reprogramming in HCC may uncover potential therapeutic targets. The CRSP complex, a multiprotein coactivator made up of at least 25 subunits, serves a critical function in transcriptional control of eukaryotic genes. It functions as a key linkage for transcription by connecting promoters to fundamental transcription machinery [5]. Among them, *CRSP8* is gaining recognition as a gene associated with lipid metabolism, playing a crucial role in attracting transcriptional mediators to specific target genes [6]. Two studies have associated *CRSP8* with melanoma and thyroid cancer; however, its particular role and mechanism in the progression of HCC and energy metabolism are still mostly unclear [10–12]. Consequently, we examined the functional properties of CRSP8 to evaluate its contribution to carcinogenesis in HCC.

## Results

### High CRSP8 expression is associated with lipid accumulation and poor prognosis in HCC

**Fig. 1:**
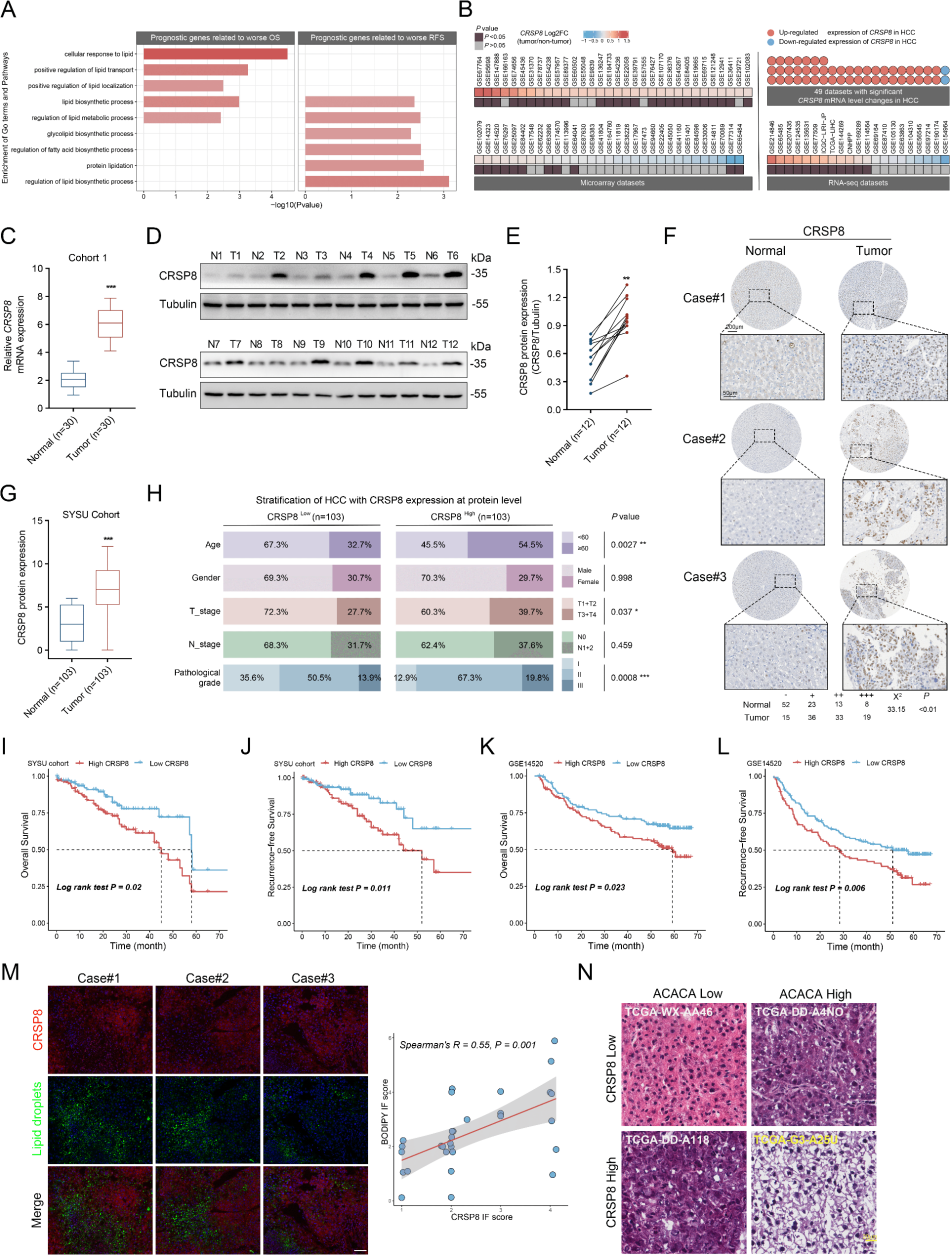
CRSP8 is upregulated in HCC, and its high expression correlates with lipid accumulation and poor prognosis in patients with HCC. **(A)** GSEA for human HCC tissues from the TCGA-LIHC cohort and GSE14520 dataset. **(B)** Extensive data mining was conducted to analyze the mRNA levels of *CRSP8* in HCC tumors compared to non-HCC tissues. **(C)** mRNA levels of *CRSP8* were confirmed by qRT-PCR analysis in 30 cases of human HCC and paired normal tissues. **(D**, **E)** Western blotting confirmed protein expression of CRSP8 in 12 human HCC and paired normal tissues. Right, quantification of gray value. **(F)** Representative IHC staining intensity of CRSP8 in HCC and normal tissues from SYSU Cohort. Scale bar, 200 μm (top); 50 μm (bottom). **(G)** CRSP8 expression in the SYSU Cohort. **(H)** Proportional differences in clinicopathological factors in the CRSP8^high^ and CRSP8^low^ expression groups from the patient cohort. The *χ*^2^ test was used to evaluate the correlation between CRSP8 expression and clinical characteristics. **(I**, **J)** Kaplan–Meier curve for CRSP8 expression in the SYSU Cohort (using median cutoff). **(K**, **L)** Relationship between CRSP8 and OS and RFS of patients with HCC was evaluated using Kaplan–Meier survival analysis; analysis was performed using the Mann–Whitney *U* test. **(M)** CRSP8 expression in representative human HCC tissues; lipid content was determined using BODIPY 493/503. Scale bar, 50 μm. Right, correlation between CRSP8 expression and lipid droplet level in HCC tissues. The *P* value was generated using the Spearman’s rank correlation test. **(N)** H&E-stained sections of human HCC samples with CRSP8^Low^-expressing (top) and CRSP8^High^-expressing (bottom), alongside either ACACA^Low^ (left)- or ACACA^High^ (right)-expressing HCC in TCGA. Scale bar, 20 μm. *P < 0.05; **P < 0.01; ***P < 0.001

Essential lipid metabolism genes are frequently upregulated in HCC, and patients with high expression of these genes typically experience a poorer prognosis [13]. We analyzed publicly accessible RNA-sequencing (RNA-Seq) data [14] and observed that genes associated with worse overall survival (OS) and recurrence-free survival (RFS) that are involved in the regulation of lipid metabolism in HCC (Fig. 1A), consistent with previously published findings [15, 16]. To identify the pivotal genes underlying lipid metabolism and to elucidate their contributions to HCC development, we compiled a dataset of lipid metabolism-associated genes (LMAGs). This dataset was used to identify key genes and their corresponding regulatory networks (Fig. S1A). Among the top upregulated prognostic genes related to worse OS and RFS, *CRSP8* caught our interest due to prior reports indicating its role in regulating cancer proliferation [10]. Earlier researches have shown that *CRSP8* is significantly elevated in various tumors [10, 11], suggesting to a common role of *CRSP8* in tumor biology. Nonetheless, there is limited understanding of its functions related to lipid metabolism in tumorigenesis and the metabolic processes linked to HCC. We performed an extensive data mining analysis involving 87 datasets that included samples from HCC patients, identifying notable alterations in *CRSP8* mRNA levels across 49 of these datasets. Of these, 47 datasets revealed a significant rise in *CRSP8* mRNA levels within HCC tissues (Fig. 1B).

To verify the findings from these public datasets, we assessed *CRSP8* upregulation through quantitative reverse-transcription PCR (qRT-PCR) in Cohort 1 (30 HCC patients). Importantly, *CRSP8* was found to be upregulated by at least two-fold in 63.33% (19 out of 30) of the HCC patients (Fig. 1C; *P* < 0.001). Subsequently, to verify the elevated protein level of CRSP8, we performed western blot analysis on 12 randomly selected paired tissues from Cohort 1. Aligning with the mRNA findings, CRSP8 protein levels were elevated in 83.33% (10 out of 12) of HCC tissues compared to adjacent tissues (Fig. 1D-E; *P* < 0.01). Furthermore, we conducted immunohistochemistry (IHC) on a tissue microarray to assess CRSP8 levels in an enlarged cohort of 103 HCC patients (SYSU Cohort). The representative staining outcomes are displayed in Fig. 1F. CRSP8 levels were significantly higher in HCC tissues compared to peri-tumorous tissues within paired samples (Fig. 1G; *P* < 0.001). Additional studies evaluated the association between CRSP8 level and various clinicopathological parameters. Patients who were older, had poorer tumor characteristics, and exhibited higher pathological grades demonstrated significantly increased CRSP8 levels (Fig. 1H). Clinically, elevated CRSP8 expression was significantly associated to more advanced stages of clinical progression and iCluster-1 HCC samples, which correlate with unfavorable outcomes. Additionally, CRSP8 levels progressively increased throughout HCC development (Fig. S1B-D). Elevated CRSP8 expression was linked to poor OS and RFS across several HCC datasets (Fig. 1I-L).

To validate the involvement of CRSP8 in lipid metabolic processes, we performed immunofluorescent staining on human tumor tissues. High CRSP8 expression correlated with lipid accumulation (Fig. 1M). We further analyzed data from The Cancer Genome Atlas (TCGA) to investigate the correlation between CRSP8 and acetyl CoA carboxylase-1 (ACACA), as well as their impact on survival. ACACA encodes an enzyme that facilitates the conversion of acetyl-CoA to malonyl-CoA, promoting lipid synthesis, and is often upregulated in various cancers, including HCC [17, 18]. Low CRSP8 expression in combination with high ACACA expression, was associated with significantly improved OS rates compared to high expression of both ACACA and CRSP8, which was linked to the poorest prognosis for OS (Fig. S1E). Additionally, histological examination of HCC patients revealed that high CRSP8 and ACACA levels (Fig. 1N, bottom right) were linked to a distinctly different histology compared to cases exhibiting low CRSP8 expression alongside high ACACA expression (Fig. 1N, top right). HCC tumors with elevated CRSP8 and ACACA expression (Fig. 1N, bottom right) demonstrated significant cell ballooning, increased translucency, and reduced overall hematoxylin and eosin (H&E) staining compared to HCC sections with low CRSP8 and high ACACA expression (Fig. 1N, top right). It’s indicated that reduced CRSP8 levels alleviated lipid accumulation associated with high ACACA expression (Fig. 1N, top right), whereas elevated CRSP8 levels correspond to a greater lipid content in human HCC sections when paired with high ACACA expression (Fig. 1N, bottom right).

In summary, our findings suggest that elevated CRSP8 expression correlates with increased lipid accumulation, more aggressive forms of HCC, and poorer patient survival when associated with high ACACA expression. Conversely, low CRSP8 expression is indicative of reduced lipid accumulation and improved patient outcomes.

### *CRSP8* facilitates proliferation of HCC cells in vitro and in vivo

**Fig. 2:**
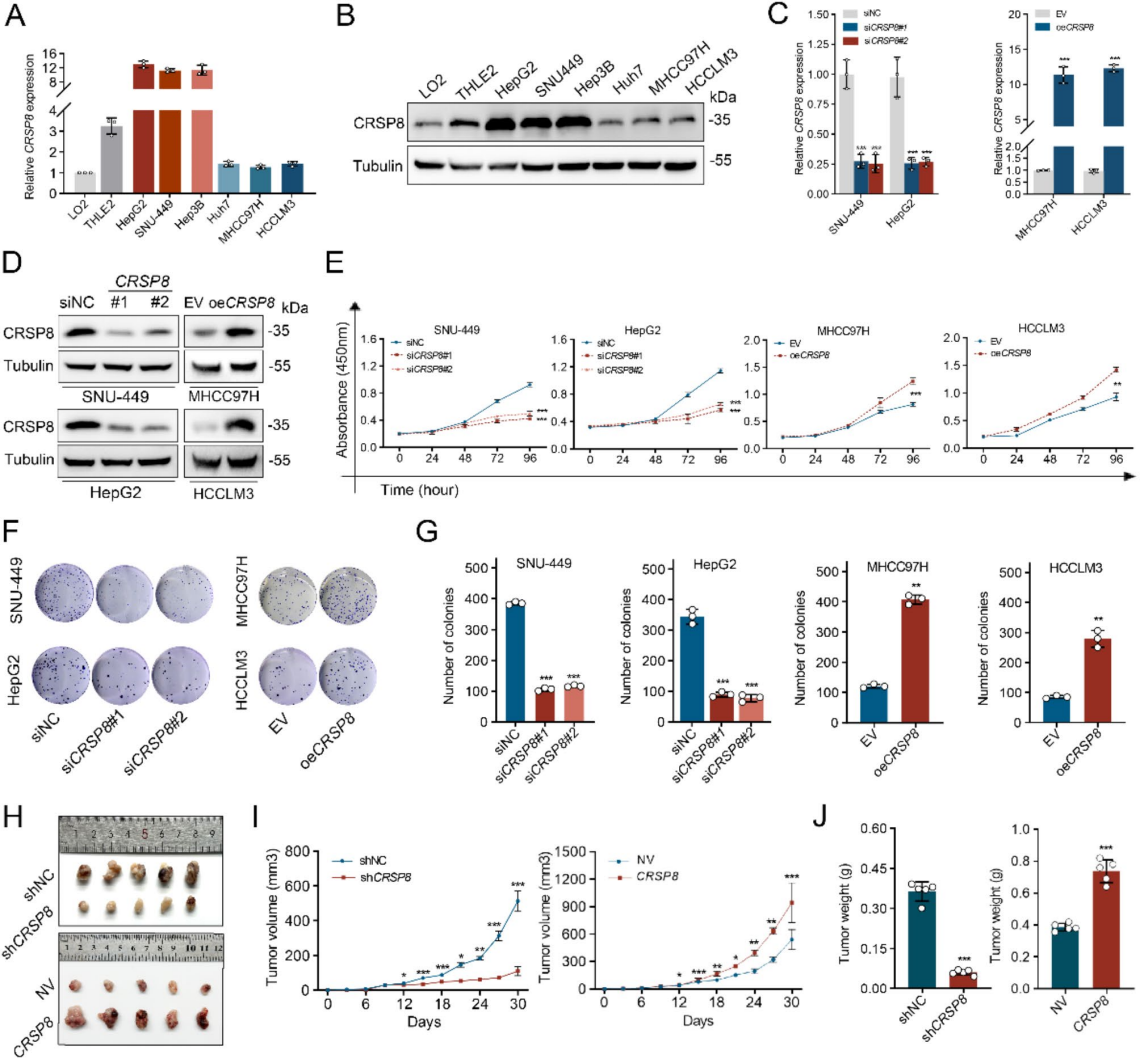
*CRSP8* facilitates HCC cell proliferation in vitro and in vivo. **(A**, **B)** qRT-PCR and western blotting analysis of CRSP8 expression in HCC cell lines and normal liver cell lines LO2 and THLE2. Tubulin was used as a loading control. **(C**, **D)** HCC cells were transfected with a mixture of *CRSP8* siRNAs and overexpression vectors. Changes in the mRNA and protein levels of CRSP8 were confirmed via western blotting and qRT-PCR analyses. **(E)** CCK-8 proliferation assay after knockdown or overexpression of *CRSP8* in HCC cells (*n* = 3). **(F)** Colony formation assay in HCC cells with knockdown or overexpression of *CRSP8*. **(G)** Quantification of the colony number. **(H**–**J)** Xenograft tumor growth of HepG2 cells with stable knockdown or overexpression of *CRSP8* (*n* = 5). (**H.**) Representative images of subcutaneous xenografts. (**I.**) Growth curves of subcutaneous xenografts (*n* = 5). (**J.**) Quantitative analysis of xenograft weight. Data are presented as mean ± SD. *P < 0.05; **P < 0.01; ***P < 0.001

CRSP8 expression was initially assessed across six HCC cells. All selected HCC cell lines exhibited increased levels of CRSP8 mRNA and protein when compared to the hepatocyte cell lines LO2 and THLE2 (Fig. 2A and B). To investigate the impact of CRSP8 on HCC growth, we conducted *CRSP*8 knockdown in HepG2 and SNU-449 cells while overexpressing *CRSP8* in MHCC97H and HCCLM3 cells (Fig. 2C-D). Cell viability was assessed using the Cell Counting Kit-8 (CCK-8) assay. The knockdown of endogenous *CRSP8* significantly reduced cell proliferation, whereas exogenous overexpression of *CRSP8* markedly increased it (Fig. 2E). Additionally, the colony formation assay demonstrated that CRSP8 knockdown inhibited the colony-forming capacity of HCC cells, while its overexpression enhanced colony formation (Fig. 2F-G). These results were further validated by EdU proliferation assay (Fig. S2A-D). Furthermore, stable knockdown of *CRSP8* significantly decreased both the weight and volume of xenograft tumors compared to controls, whereas exogenous overexpression of *CRSP8* significantly enlarged tumor volume and weight (Fig. 2H-J). Collectively, these findings suggest that *CRSP8* activates HCC proliferation both in vitro and in vivo.

The involvement of CRSP8 in HCC metastasis was evaluated by investigating its impacts on cell migration and invasion. Transwell and wound-healing assays revealed that silencing *CRSP8* markedly reduced the migration and invasion capabilities of HCC cells (Fig. S2E-F). Conversely, *CRSP8* overexpression greatly enhanced both migration and invasion (Fig. S2G-H). Given that the epithelial-to-mesenchymal transition (EMT) plays an essential role in tumor invasion and metastasis [19, 20], we further investigated the impact of CRSP8 on EMT. Silencing *CRSP8* resulted in reduced levels of EMT inducer Snail and mesenchymal markers N-cadherin and β-catenin, while concurrently increasing the expression of the epithelial marker E-cadherin. In contrast, *CRSP8* overexpression elicited the opposite effects (Fig. S2E). Overall, these findings underscore the impact of *CRSP8* in facilitating metastasis in HCC cells.

### *CRSP8* regulates fatty acid metabolism pathways

**Fig. 3:**
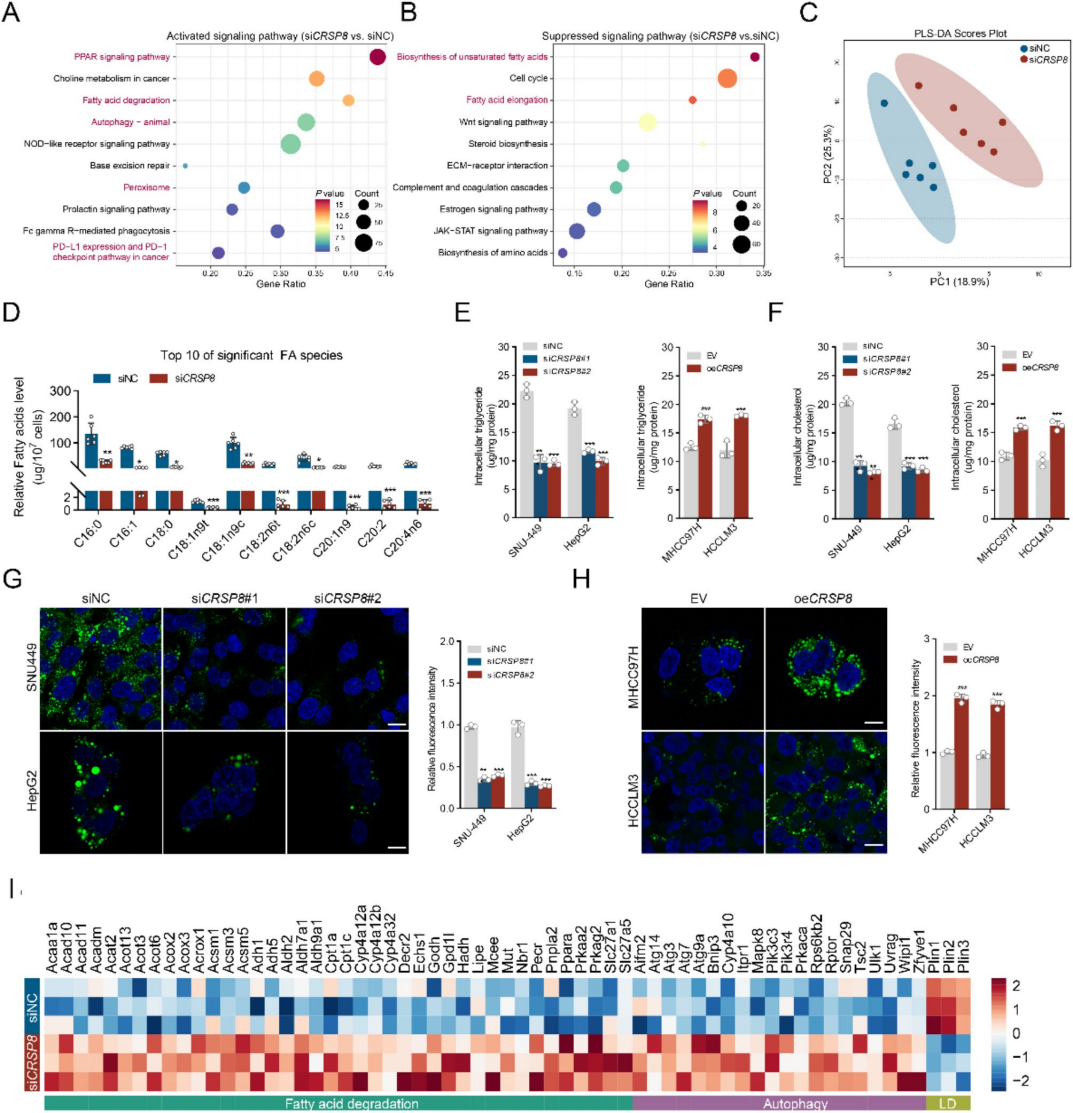
*CRSP8* regulates fatty acid metabolism reprogramming in HCC cells. **(A**, **B)** Left, activated signaling pathways identified using GSEA with the hallmark gene set in RNA-seq data from SNU-449 cells with *CRSP8* knockdown. Right, GSEA results of the indicated suppressed pathways in SNU-449 cells with *CRSP8* knockdown. **(C)** Inherent clustering between control and si*CRSP8* groups visualized using the principal component analysis. **(D)** Intracellular free fatty acids in the control and si*CRSP8* groups determined using GC-MS. Data are representative of six independent experiments. The data were analyzed using the student’s *t*-test. **(E)** Cellular content of triglycerides in HCC cells with knockdown or overexpression of *CRSP8*. **(F)** Cellular content of cholesterol in HCC cells with knockdown or overexpression of *CRSP8*. **(G**, **H)** Neutral lipids stained with BODIPY 493/503 in HCC cells. Scale bar, 10 μm. **(I)** Heatmap of downregulated and upregulated genes in RNA-seq data from SNU-449 cells with *CRSP8* knockdown. Data are presented as mean ± SD. *P < 0.05; **P < 0.01; ***P < 0.001

Transcriptomic and metabolomic analyses were conducted to identify potential signaling pathways modulated by *CRSP8*. We initiated our investigation with RNA-seq on SNU-449 cells following *CRSP8* knockdown. Results from gene set enrichment analysis (GSEA) indicated that *CRSP8* knockdown affects pathways related to FA metabolism and autophagy. Specifically, autophagy and fatty acid degradation pathways (involving peroxisome proliferator-activated receptors, PPARs and peroxisome) were identified as the two primary activated pathways (Fig. 3A-B).

Next, lipidomics analysis using GC/TOF-MS was conducted on SNU-449 cells transfected with either *CRSP8* siRNA or scrambled siRNA (*n* = 6 per group). Principal component analysis was employed to illustrate the natural clustering of the control and experimental groups (Fig. 3C), revealing a clear distinction that suggested significant variation in lipid composition between the two groups. Additionally, metabolite analysis showed that *CRSP8* knockdown resulted in a decrease in FFA contents (Fig. 3D), suggesting that CRSP8 may be essential for modulating lipid metabolism in HCC. To further confirm these observations, we analyzed intracellular lipid contents. The levels of intracellular TGs and cholesterols were substantially reduced in cells with *CRSP8* silencing, whereas *CRSP8* overexpression led to an elevation in lipid contents (Fig. 3E-F). Furthermore, neutral lipids stained with BODIPY 493/503 showed reduced fluorescence following *CRSP8* knockdown (Fig. 3G), while exogenous overexpression of CRSP8 markedly increased fluorescence (Fig. 3H). Heat maps derived from the transcriptomic analysis showed a downregulation of both FA degradation and autophagy pathways, along with an upregulation of LD pathway in *CRSP8* knockdown groups (Fig. 3I). Collectively, these findings suggest that *CRSP8* plays a critical role in regulating lipid metabolism within HCC cells.

### *CRSP8* suppressed lipophagy and FAO in HCC cells

An increase in cellular lipid content may result from enhanced lipid biosynthesis, elevated FA uptake, and reduced lipid catabolism. Consequently, we evaluated the expression levels of key genes associated with FA synthesis (*SREBP1*, *FASN*, *ACACA*, *ACLY*, *SCD1*, *MLYCD*, *ACSL1*, *ACSL3*, *ACSL5*, and *FABP5*), cholesterol biosynthesis (*HMGCR* and *SREBP2*), FA uptake (*CD36*), and FAO (*CPT1A* and *ACOX1*) using RNA-seq data from *CRSP8* knockdown SNU-449 cells (Fig. 3I and Fig. S3A). Notably, we found that mRNA and protein levels of ACLY, ACACA, FABP5, and CD36 were significantly reduced in cells with *CRSP8* silencing, whereas these levels increased in cells with *CRSP8* overexpression (Fig. S3B-E). In the TCGA cohort, a consistent positive correlation was identified between *CRSP8* expression and lipogenic enzymes in liver cancer (Fig. S3F). These observations suggest that CRSP8 facilitates de novo FA synthesis by upregulating these lipogenic enzymes.

Pathways associated with autophagy and FA degradation (involving PPARs and peroxisome) emerged as the two leading pathways through GSEA (Fig. 3A). The qRT-PCR analysis of gene expression also indicated an increase in autophagy and FAO while showing a reduction in LD formation upon *CRSP8* knockdown (Fig. S4A). We hypothesized that elevated levels of CRSP8 may hinder autophagy and FA degradation through the PPARs pathway [21–23].

**Fig. 4:**
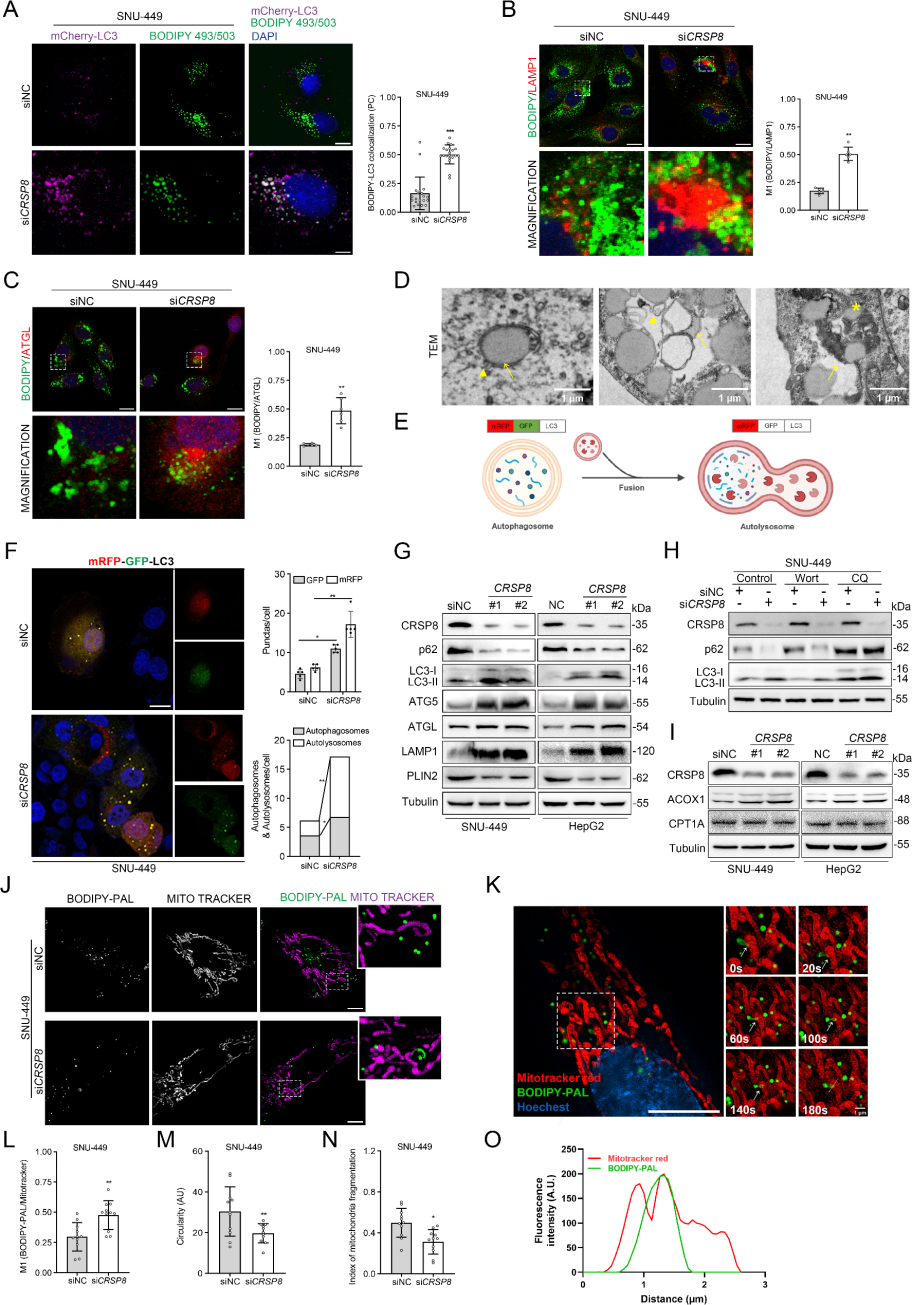
*CRSP8* knockdown-activated lipophagy and FAO in HCC cells. **(A)** Colocalization of BODIPY 493/503-stained lipid droplets and mcherry-LC3 in the control and si*CRSP8* group of SNU-449 cells (PC = Pearson coefficient). Nuclei are stained with DAPI. Scale bar, 10 μm. **(B**, **C)** Representative co-staining of BODIPY/LAMP1 and BODIPY/ATGL in SNU-449 cells. Nuclei were stained with DAPI. Scale bar, 10 μm. **(D)** Direct association of autophagosomes with LDs observed in electron micrographs of SNU-449 cells with *CRSP8* knockdown. Arrowheads, autophagosomes; arrows, LDs; asterisks, autolysosomes. Cells were fixed with 3% glutaraldehyde in phosphate buffer; ultrathin sections (70 nm thick) were obtained using a microtome (Leica EM UC6) and examined using a transmission electron microscope as described in the methods. **(E)** Schematic diagram of autophagic flux tracked using the mRFP-GFP-LC3 reporter. **(F)** Representative images and quantification of autophagic flux in SNU-449 cells (*n* = 5 per group). Scale bar, 10 μm. **(G)** Representative blots for indicated proteins indicating lipophagy and lipolysis levels in SNU-449 and HepG2 cells with *CRSP8* knockdown. **(H)** Western blot analysis of CRSP8, p62, and LC3 expression in control and si*CRSP8* SNU-449 cells treated with or without wortmannin (Wort) or chloroquine (CQ). **(I)** Western blot analysis demonstrating the effects of *CRSP8* silencing on the expression of enzymes critical for FAO. **(J**-**O)** Representative super resolution live images (**J**) of the control and si*CRSP8* SNU-449 cells pulsed with BODIPY-PAL (overnight incubation) and imaged after a 24 h chase period. Scale bar, 10 μm. Mitochondria labeled with MitoTracker deep red (30 min) before imaging, colocalization index M1 (**L**), mitochondrial circularity (**M**), and index of mitochondrial fragmentation (number of mitochondria/total mitochondrial area) (**N**). Zoomed boxed areas show colocalization of the mitochondria (magenta) with BODIPY-PAL-signals (green). A representative time-lapse montage of BODIPY-PAL-stained FAs and MitoTracker deep red-labeled mitochondria in live cells (**K**). Scale bar, 10 μm. Linescan analysis colocalizing BODIPY-PAL with mitochondria (**O**) from SNU-449 cells with *CRSP8* knockdown. Mean ± SD, *n* = 3 biological replicates analyzed using unpaired the student’s *t*-test (two-tailed) or Mann–Whitney test (two-tailed), *P < 0.05; **P < 0.01; ***P < 0.001

To investigate whether lipophagy is essential for maintaining LD homeostasis, we transiently expressed mCherry-LC3 plasmids in control groups while staining with BODIPY 493/503 to further verify the association between autophagic machinery components and lipid droplets. Following *CRSP8* knockdown, we observed a notable increase in the colocalization of BODIPY 493/503 and mCherry-LC3 (Fig. 4A and Fig. S4B). And immunofluorescence analysis demonstrated a marked reduction in lipid droplets (indicated by BODIPY-positive dots) alongside enhanced lipophagy (evidenced by BODIPY/LAMP1 colocalization) and increased lipolysis (shown by BODIPY/ATGL colocalization) following *CRSP8* knockdown (Fig. 4B-C and Fig. S4C-D). To more accurately assess lipophagy progression, we conducted transmission electron microscopy (TEM) and observed double-membrane vesicles resembling autophagosomes (indicated by arrowheads) surrounding LDs (marked by arrows), as well as degradative structures enriched in LDs (asterisks, Fig. 4D and Fig. S4E), consistent with previous reports on lipophagy [7]. Given that lipophagy is related to autophagy, we investigated whether changes in *CRSP8* expression influence autophagosomes functions. We transfected HepG2 and SNU-449 cells with a dual fluorescent mRFP-GFP-LC3 plasmid reporter, which allows monitoring of autophagosomes maturation into autolysosomes due to the pH sensitivity of GFP (Fig. 4E). As illustrated in Fig. 4F and Fig. S4F, *CRSP8* knockdown resulted in an increase in both autophagosomes (mCherry+, GFP+) and autolysosomes (mCherry+, GFP-). This finding was further validated by western blot analysis, indicating signs of activated autophagy (reflected by elevated levels of LC3-II, ATG5, and LAMP1 along with reduced P62), increased lipolysis (suggested by higher ATGL levels), and enhanced FAO (indicated by increased ACOX1) (Fig. 4G). Moreover, when these stable cells were exposed to the autophagy inhibitors wortmannin (Wort) and chloroquine (CQ), Wort prevented the degradation of p62 and the conversion of LC3 induced by *CRSP8* knockdown. In contrast, after CQ treatment, cells with stable *CRSP8* knockdown exhibited slightly elevated levels of LC3-II while maintaining similar levels of p62 compared to control vector cells (Fig. 4H and Fig. S4G). These results reinforce the notion that CRSP8 negatively regulates autophagy and lipophagy flux in HCC cells.

FAs are stored as TGs in LDs. These droplets consist of a neutral lipid core encased in a phospholipid monolayer interspersed various proteins, which are dynamically modified to satisfy energy demands and promote FAO [24]. qRT-PCR analysis also revealed enhanced FAO following *CRSP8* knockdown (Fig. S4A). We hypothesized that the degradation of LDs through autophagy could provide FAs to support mitochondrial FAO [25].

Initially, we analyzed the protein levels of corresponding markers. Western blotting suggested activated FAO (as demonstrated by increased ACOX1 levels) in response to *CRSP8* knockdown (Fig. 4I). Subsequently, we monitored FAs incorporation into LDs and their spatial relationship with the mitochondrial network through live imaging with the saturated 16-carbon fluorescent analog BODIPY-palmitate (BODIPY-PAL). This compound was utilized in lipid trafficking studies as it integrates into neutral lipids found in lipid droplets [26]. In HCC cells with *CRSP8* knockdown, BODIPY-PAL colocalized with the mitochondrial network throughout the pulse-chase period (Fig. 4J; Movies S1 and S2), indicating efficient transfer of FA to mitochondria. Representative dynamic images over time are displayed in Fig. 4K and O. Previous studies have emphasized the importance of an interconnected mitochondrial network for effective FAs absorption [27–30]. HCC cells with impaired autophagy exhibited characteristics of fragmented mitochondria, evidenced by elevated mitochondrial circularity and a higher fragmentation index (the ratio of mitochondria number to total mitochondrial area). In HCC cells with *CRSP8* silencin*g*, these indices improved significantly (Fig. 4L-N). Mitochondrial dynamics and morphology serve as crucial indicators of mitochondrial metabolic and bioenergetic conditions [31]. CPT1A catalyzes the conversion of long-chain fatty acyl-CoA into their respective fatty acyl-carnitines, facilitating transport into mitochondrial matrix and representing as a critical step in FAO. We assessed FAO-related oxygen consumption by measuring the oxygen consumption rates (OCR) under conditions treated with etomoxir, a CPT1A inhibitor. In HepG2 cells, after brief treatment with etomoxir, both maximal respiration and spare respiratory capacity decreased to levels comparable to those observed in autophagy-deficient cells treated with wortmannin. In HCC cells with *CRSP8* knockdown, these two parameters showed significantly improvement.

Considering that impairments in lipophagy can hinder the delivery of FAs to mitochondria, we investigated the impact of CRSP8 inhibition on mitochondrial FAO by measuring [9,10–3 H]-palmitate oxidation rates. Silencing *CRSP8* notably enhanced the capacity of HCC cells for FAO. Additional ATP level analysis revealed similar trends. These results suggest that FAO may operate downstream of lipophagy, with lipophagy mobilizing FAs by degrading LDs, thereby sustaining mitochondrial FAO in HCC cells. LysoTracker staining further indicated that autophagy flux increased following *CRSP8* knockdown, similar to rapamycin treatment (Fig. S6A-B).

### Effect of *CRSP8* on lipophagy and FAO is dependent on hepatic nPPARα

**Fig. 5:**
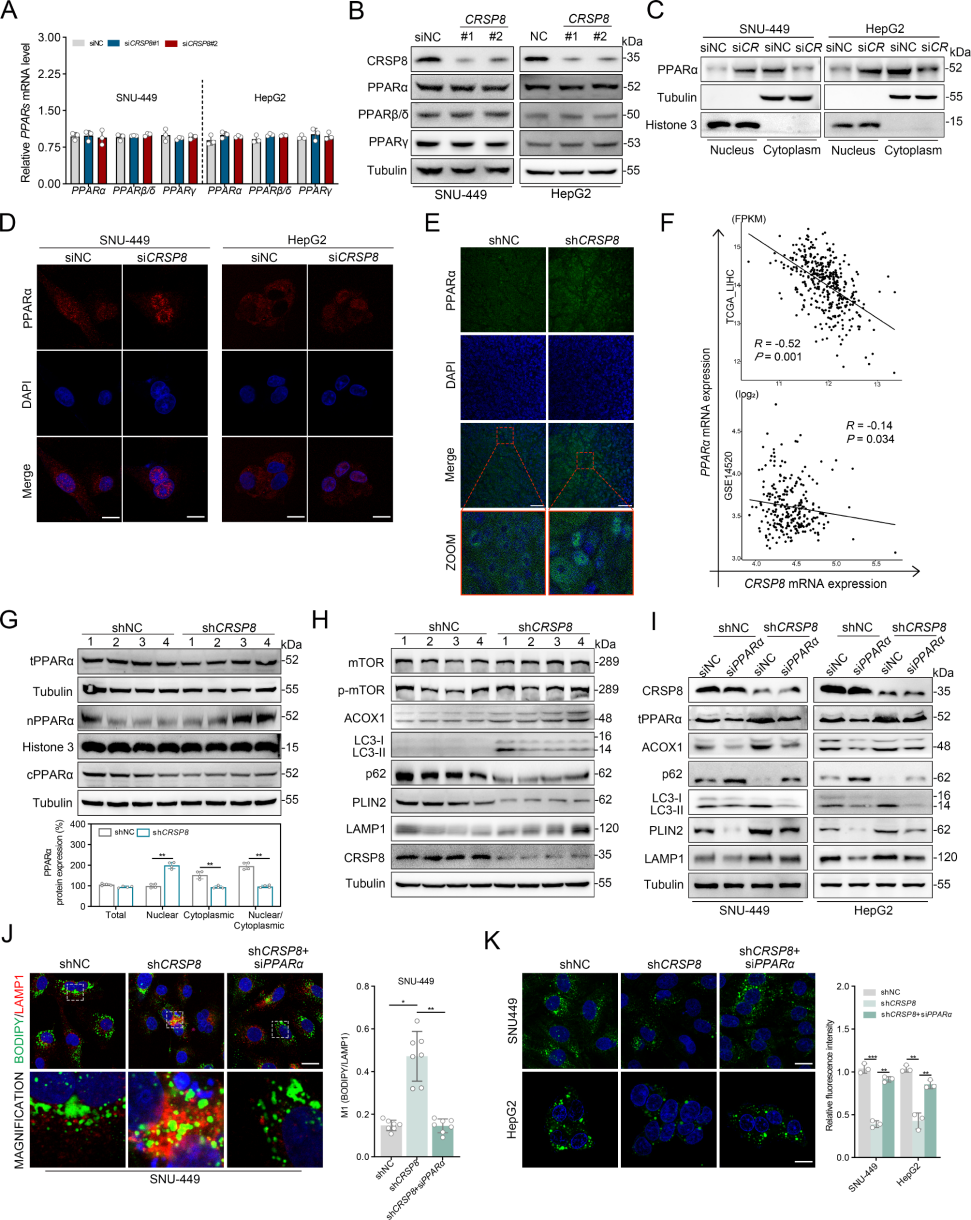
Impaired lipophagy and FAO induced by *CRSP8* is dependent on hepatic nPPARα. **(A**, **B)** Validation of the expression of PPARs in SNU-449 and HepG2 cells with *CRSP8* knockdown using qRT-PCR and western blot analyses. Mean ± SD, *n* = 3 biological replicates analyzed using unpaired Student’s *t*-test (two-tailed). **(C)** PPARα expression in cytoplasmic and nuclear fractions, detected using immunoblot analysis. Tubulin and Histone 3 were used as loading controls for the cytoplasmic and nuclear fractions, respectively. **(D)** PPARα (red) expression in indicated SNU-449 and HepG2 cells, detected using an immunofluorescence assay. The merged images show overlays of PPARα (red) and nuclear staining with DAPI (blue). Scale bar, 10 μm. **(E)** Immunofluorescence staining of PPARα (green), Scale bar, 50 μm. **(F)** Pearson correlation coefficient between *CRSP8* and *PPARα* in the GSE14520 (*n* = 242) and TCGA-LIHC (*n* = 364) cohorts. **(G**-**I)** Xenograft tumor samples from BALB/c nude mice with *CRSP8* knockdown. (**G.**) Promotion of the PPARα nuclear accumulation by *CRSP8* silencing (*n* = 4 per group). (**H.**) Protein expressions of indicated proteins of lipophagy in the liver (*n* = 4 per group). (**I.**) Western blot analysis of proteins indicating lipophagy and FAO in SNU-449 and HepG2 cells. Tubulin was used as a loading control. **(J)** Representative co-staining of BODIPY/LAMP1 in SNU-449 cells with the indicated treatment. Nuclei were stained with DAPI. Right, quantification of colocalization index M1 with ImageJ. Scale bar, 10 μm. Mean ± SD, *n* = 7 biological replicates. **(K)** Detection of neutral lipids using BODIPY 493/503 staining in SNU-449 and HepG2 cells with the indicated treatment. Right, quantification of fluorescence intensity with ImageJ. Scale bar, 10 μm. Mean ± SD, *n* = 3 biological replicates analyzed using unpaired student’s *t*-test (two-tailed), *P < 0.05; **P < 0.01; ***P < 0.001

PPARs play a crucial role in lipid metabolism and serve as essential regulators of autophagy and FAO by activating the transcription of genes related to these processes within the nucleus [21–23]. In line with the transcriptomic data, mRNA levels of PPARα target genes exhibited a notable increase (Fig. S4A). We hypothesized that *CRSP8* may regulate genes related to lipophagy and FAO via PPARs. Consequently, we assessed the expressions levels of PPARα, PPARβ/δ, and PPARγ (Fig. 5A-B; Fig. S7A-B;). Notably, while silencing *CRSP8* did not alter the total amount of PPARα protein, it significantly enhanced the nuclear-to-cytoplasmic ratio, which was diminished upon *CRSP8* enrichment (Fig. 5C; Fig. S7C). As a transcription factor, the function of PPARα heavily depends on its localization within the nucleus, a process that is regulated by the dynamic shuttling of this protein between the cytoplasm and the nucleus. Immunofluorescence analysis of HCC cells and the subcutaneous xenograft model further confirmed these findings (Fig. 5D-E; Fig. S7D). Correlation analysis revealed a positive relationship between *CRSP8* and *PPARα* in both the TCGA cohort and GSE14520 (Fig. 5F). Western blot analysis of the subcutaneous xenograft model demonstrated that *CRSP8* knockdown led to a significant increase in nuclear PPARα levels while reducing cytoplasmic PPARα levels, without altering the total protein content (Fig. 5G). Consistent with the findings in HCC cells, the protein levels of PPARα targets were markedly elevated, independent of any changes in the upstream mTOR signaling pathway (Fig. 5H).

To further validate that *CRSP8* influences lipophagy and FAO by modulating the dynamic movement of PPARα between the cytoplasm and nucleus, we reduced *PPARα* expression in cells where *CRSP8* was silenced. The knockdown of *PPARα* notably eliminated the increase in LC3, PLIN2, LAMP1, and ACOX1 levels induced by *CRSP8* silencing (Fig. 5I). Moreover, immunofluorescence analysis indicated that *PPARα* knockdown suppressed the increased lipophagy caused by *CRSP8* knockdown in both SNU-449 and HepG2 cells (Fig. 5J; Fig. S7E), and also increased the levels of intracellular neutral lipids (Fig. 5K). Similar trends were observed for FAO and ATP levels (Fig. S7F-I). Consequently, evidence from both cellular and animal studies indicated that *CRSP8* inhibits the nuclear localization of PPAR, thereby impairing PPARα-mediated lipophagy and FAO, which ultimately leads to increased lipid accumulation.

### *CRSP8* represses nuclear translocation of PPARα via interaction with RAN and CRM1

**Fig. 6:**
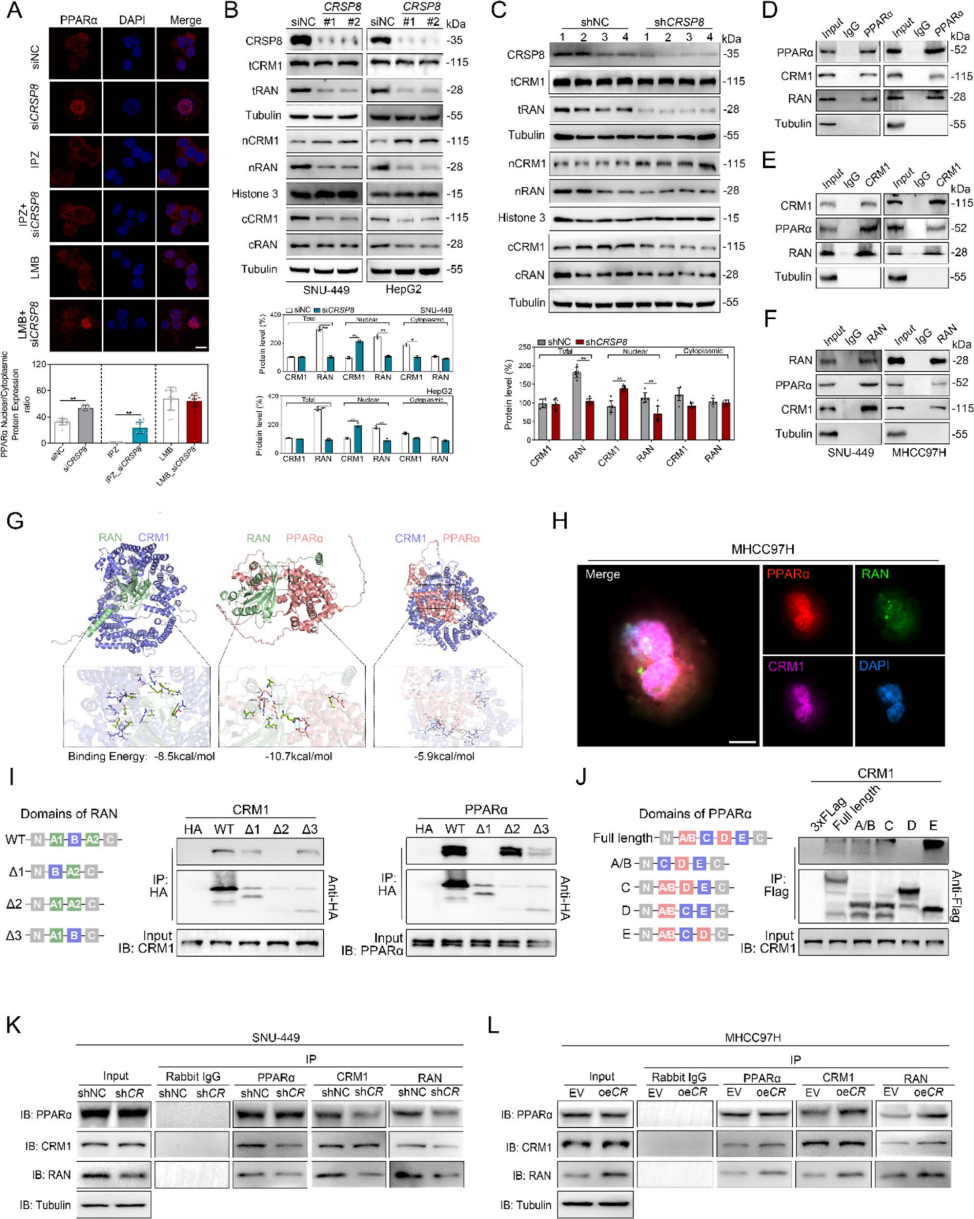
*CRSP8* regulates nuclear translocation of PPARα through the RAN-CRM1-PPARα export heterotrimer. **(A)** Immunofluorescence of PPARα with importazole (IPZ, 10µM) and leptomycin B (LMB, 10ng/µL) treatment of HepG2 cells with or without *CRSP8* knockdown. HepG2 cells were pretreated with IPZ for 24 h or with LMB for 12 h (Scale bar, 10 μm). Differences between groups were determined using unpaired two-tailed student’s *t*-test (representative images are shown from one of three biologically independent experiments for each condition). **(B)** Total, nuclear, and cytoplasmic protein levels of CRM1 and RAN with *CRSP8* knockdown in SNU-449 and HepG2 cells (*n* = 3). Differences between groups were determined using unpaired two-tailed Student’s *t*-test. The findings were confirmed in two independent experiments. **(C)** Total, nuclear, and cytoplasmic protein levels of CRM1 and RAN in the xenograft tumor of mice treated as in Fig. . 2H (*n* = 4 per group). Differences between groups were determined using one-way ANOVA test followed by Tukey’s multiple comparison. Data are presented as mean values ± SD. The average of gene and protein expression levels in the control group is normalized as 100%. **(D**-**F)** Interaction of endogenous PPARα with CRM1 and RAN determined using western blot analysis following coimmunoprecipitation. Tubulin was used as a nonspecific control. **(G)** Interactions between PPARα and RAN, CRM1 and RAN, and PPARα and CRM1 predicted using RosettaDock. **(H)** Colocalization of PPARα, CRM1, and RAN detected using confocal microscopy of MHCC97H cells. Scale bar, 10 μm. **(I**-**J)** Representative immunoblots of the various constructs of RAN-HA and PPARα-Flag in a coIP assay of HepG2 cells. *n* = 3 technical replicates per group. The A1, B, and A2 domains of RAN are 10-47AA, 48-125AA, and 126-209AA, respectively. The A/B, C, D and E domains of PPARα are 13-101AA, 102-166AA, 167-244AA, and 245-439AA, respectively. **(K**-**L)** Coimmunoprecipitation of PPARα, CRM1, and RAN in indicated SNU-449 (*CRSP8* knockdown) and MHCC97H (*CRSP8* overexpression) cells, detected using immunoblot analysis

The movement of PPARα between the nucleus and cytoplasm is precisely regulated by importins and exportins [32, 33]. The application of importazole (IPZ) to inhibit the nuclear import receptor led to the accumulation of PPARα in the cytoplasm; however, *CRSP8* knockdown still facilitated the nuclear accumulation of PPARα, even under IPZ treatment. Conversely, inhibiting the nuclear export receptor with leptomycin B (LMB) resulted in increased levels of PPARα in the nucleus, although combining LMB treatment with *CRSP8* knockdown did not further elevate nuclear PPARα levels (Fig. 6A). These findings imply that *CRSP8* might contribute to modulating the nuclear export of PPARα rather than its translocation into the nucleus.

Chromosome maintenance region 1 (CRM1) and the small GTPase RAN, a member of the *ras* family, are essential for the nuclear export of proteins, as they bind to cargo proteins to form an export heterotrimer [32–35]. RAN is known to form a complex with CRM1 to mediate the nuclear export of PPARα [36]. To investigate whether *CRSP8* facilitates the nuclear export of PPARα through modulation of the RAN-CRM1-PPARα complex, we examined the interactions among RAN, CRM1, and PPARα in HCC. In HCC cells with *CRSP8* knockdown, nuclear CRM1 levels significantly increased, while cytoplasmic CRM1 levels decreased without affecting total protein levels. Conversely, the concentrations of RAN in both nucleus and cytoplasm declined accompanied by a reduction in total protein levels (Fig. 6B). Consistent with in vitro results, CRM1 protein expression in the nucleus was elevated in the control-treated mice compared to the *CRSP8* knockdown group while cytoplasm levels were reduced. Furthermore, RAN expression was decreased following *CRSP8* knockdown (Fig. 6C). Collectively, these findings indicated that *CRSP8* knockdown enhances PPARα nuclear localization by inhibiting RAN/CRM1-mediated nuclear export. Their interactions among CRM1, RAN, and PPARα were further confirmed through western blot analysis following coimmunoprecipitation (co-IP) assays. RAN was shown to interact with both CRM1 and PPARα in hepatoma cells (Fig. 6D-F). RosettaDock analysis also predicted the interaction of RAN with CRM1 and PPARα (Fig. 6G). Moreover, notable colocalization of CRM1, RAN, and PPARα was observed (Fig. 6H), further supporting their association. A positive association was noted among the levels of *CRM1*, *RAN*, and *PPARα* in the TCGA cohort (Fig. S8A).

To further investigate which domain of RAN and PPARα is crucial for the binding with CRM1, various RAN and PPARα truncations were transfected into HepG2 cells (Fig. 6I-J). The RAN protein is composed of N, A1, B, A2, and C domains from the N terminus to the C terminus. Based on the domains present in RAN, mutants of RAN, with truncating mutations in the A1 (Δ1), B (Δ2), or A2 (Δ3) domains, were constructed for IP assays. We found that RAN mutants with truncating mutation in the B domain no longer interacted with CRM1, and with truncating mutation in the A1 domain no longer interacted with PPARα (Fig. 6I). The PPARα protein is composed of N, A/B, C, D, E and C domains from the N terminus to the C terminus. Based on the domains present in PPARα, mutants of PPARα, with truncating mutations in the A/B, C, D or E domains, were constructed for IP assays. We found that PPARα mutants with truncating mutation in the D domain no longer interacted with CRM1 (Fig. 6J). These findings revealed that RAN regulated the translocation and activation of the PPARα signaling pathway.

Interestingly, overexpression of *CRSP8* enhanced the interactions within the RAN-CRM1-PPARα complex, whereas *CRSP8* knockdown repressed these interactions (Fig. 6K-L). Together, these findings indicate that *CRSP8* attenuates the nuclear localization of PPARα by inhibiting RAN/CRM1-mediated nuclear export.

### *CRSP8* regulates the nucleus–cytoplasm shuttling heterotrimer via transcriptionally activating *RAN*

**Fig. 7:**
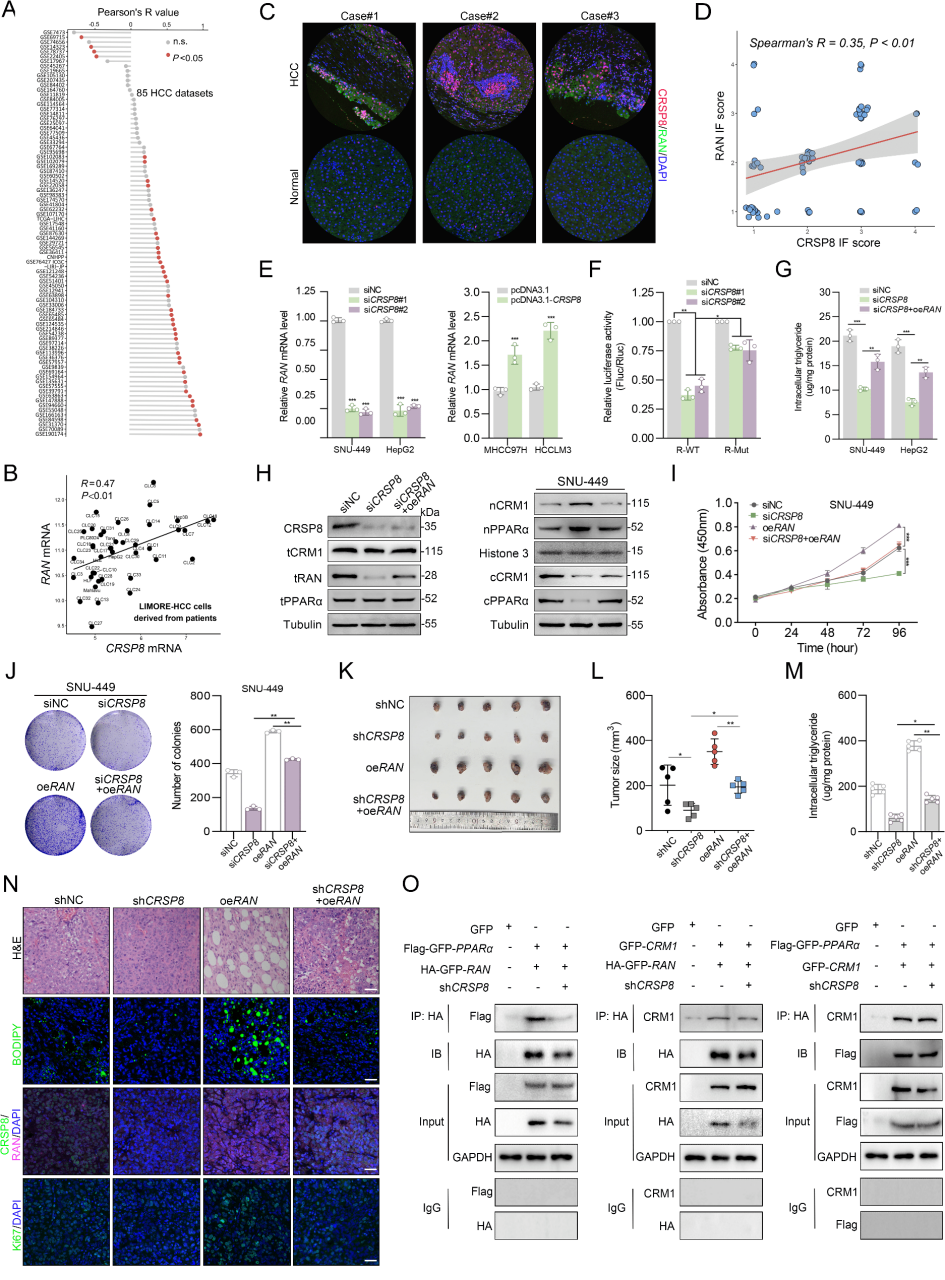
*RAN* is a functionally important target of *CRSP8* in HCC. (**A)** Correlation between mRNA levels of *CRSP8* and *RAN* in tumor tissues analyzed using 85 HCC datasets. **(B)** Analysis of the correlation between *CRSP8* and *RAN* using public data obtained from LIMORE. **(C**, **D)** CRSP8 and RAN expressions in control and tumor tissues from patients with HCC analyzed using immunofluorescence staining; a positive correlation between these proteins is shown (right). **(E)** Effect of *CRSP8* manipulation on *RAN* expression assessed using qRT-PCR. **(F)** Reduction in the luciferase activity of the *RAN* promoter by *CRSP8* knockdown. **(G)** Triglyceride content of SNU-449 and HepG2 cells. **(H)** Total, nuclear, and cytoplasmic levels of CRM1 and PPARα in *CRSP8*-knockdown cells transfected with the *RAN-*overexpression plasmid analyzed using immunoblotting. **(I)** Proliferation of SNU-449 cells with *CRSP8* knockdown or *RAN* overexpression, analyzed using the CCK-8 assay (*n* = 3). **(J)** Colony formation assay for SNU-449 cells with *CRSP8* knockdown or *RAN* overexpression. Right, quantification of the colony number. **(K**, **L)** Xenograft tumor growth of HepG2 cells with stable *CRSP8* knockdown or *RAN* overexpression (*n* = 5). **K** Representative images of subcutaneous xenografts. **L** Quantitative analysis of xenograft size. **(M)** Triglyceride content in harvested xenograft tumors. **(N)** Representative images of tumors from each group; CRSP8, RAN, and Ki67 expression in the tumor tissues was assessed using immunofluorescence staining. BODIPY 493/503 was used to detect the accumulation of lipid droplets in frozen sections of the tumor tissues. Scale bar, 50 μm. **(O)** Association of HA-GFP-*RAN* and Flag-GFP-*PPARα*, HA-GFP-RAN and GFP-*CRM1*, and Flag-GFP-*PPARα* and GFP-*CRM1* in HEK293T cells with or without stable *CRSP8* knockdown analyzed using the coimmunoprecipitation assay. Data are presented as mean values ± SD. Differences between groups were determined using unpaired two-tailed Student’s *t*-test

We found that silencing *CRSP8* did not significantly change the overall levels of CRM1 and PPARα proteins; however, it did enhance the total amount of RAN protein. Notably, the levels of RAN protein were in agreement with *CRSP8* levels. To elucidate the molecular mechanism by which CRSP8 regulates in nuclear accumulation of PPARα and RAN, we initially examined publicly available data and observed a positive relationship between *CRSP8* and *RAN* in various HCC datasets and cell lines (Fig. 7A-B). Furthermore, immunofluorescence analysis of the SYSU cohort validated comparable expression patterns for CRSP8 and RAN (Fig. 7C-D). Alterations in *CRSP8* levels also associated with changes in the mRNA levels of *RAN* in HCC cells (Fig. 7E). Building on these observations, we proposed that the transcription factor *CRSP8* enhances the formation of RAN-CRM1-PPARα heterotrimer by directly increasing the transcriptional activity of *RAN*, thereby facilitating the nuclear-cytoplasmic shuttling of PPARα. Given the role of *CRSP8* in transcriptional regulation, we conducted a luciferase reporter assay to investigate how it affects the promoter activity of *RAN*. It revealed that *CRSP8* enhanced the luciferase activity associated with the *RAN* promoter, suggesting an interaction with the *RAN* promoter (Fig. 7F).

We also assessed the impact of *RAN* on lipogenesis. As shown in Fig. 7G, *RAN* overexpression rescued the intracellular TG levels that were downregulated by sh*CRSP8* in HCC cells. Comparable effects were noted with BODIPY493/503 staining (Fig. S8B). To further ascertain that *CRSP8* regulates the nuclear-cytoplasmic shuttling of the heterotrimer via *RAN*, we conducted a plasmid for *RAN* overexpression (pcDNA3.1-*RAN*) and verified that the successful overexpression of *RAN* restored the shuttling capacity of CRM1 and PPARα, which were disrupted by *CRSP8* knockdown via western blotting experiments. This indicates that *CRSP8* may function upstream of *RAN* to regulate the interaction among the components of the RAN-CRM1-PPARα complex (Fig. 7H; Fig. S8C). Additionally, CCK-8 and colony formation experiments demonstrated *RAN* overexpression notably reversed the effects of sh*CRSP8* on cell proliferation (Fig. 7I-J; Fig. S8D-E). To further substantiate the role of *RAN* in mediating the regulatory function of *CRSP8*, we employed a xenograft model. The subcutaneous xenograft model indicated that *RAN* overexpression significantly enhanced tumor growth following *CRSP8* knockdown (Fig. 7K-L). The reduction in TG content was also counteracted by the upregulation of *RAN* in tumors with stable *CRSP8* knockdown (Fig. 7M). When compared to the control group, the *RAN*-overexpressing group displayed significant cell ballooning, increased translucency, and reduced overall H&E staining (Fig. 7N). Tumors derived from the *CRSP8* knockdown group exhibited lower Ki67 expression compared to those from the control group, and *RAN* overexpression abrogated the effects of *CRSP8* knockdown on HCC growth. A significant positive relationship was observed between CRSP8 and Ki67 (Fig. 7N). The reduction in neural lipid content was also restored by *RAN* overexpression in tumors with stable *CRSP8* knockdown (Fig. 7N).

Subsequently, we confirmed the interaction of the RAN-CRM1-PPARα heterotrimer in HEK293T cells through co-IP assay, where we overexpressed all three component proteins. Consistent with our findings in HCC cells, *CRSP8* interfered with the interactions between RAN and PPARα as well as between RAN and CRM1. However, the binding between CRM1 and PPARα was unaffected by *CRSP8* (Fig. 7O). Overall, these results indicate *CRSP8* can regulate the RAN-CRM1-PPARα heterotrimer by transcriptionally regulating the expression of *RAN*. Our findings suggest that *RAN* plays a mediating role in the regulatory effects of *CRSP8* on HCC.

### Interfering with FA metabolism attenuated the immunosuppressive TME and improved the response to anti-PD-L1 therapy in CRSP8-enriched HCC

Previous research has shown that the accumulation of lipids and the concurrent reduction in FA catabolism impair immune monitoring by facilitating the growth of exhausted effector T cells. This dysregulation promotes hepatocyte survival and proliferation, ultimately contributing to HCC progression [37–39]. Steatotic HCC is characterized by a tumor microenvironment (TME) which is rich in immune cells but displays significant immune exhaustion, especially T cell exhaustion, along with an abundance of M2 macrophages and cancer-associated fibroblasts. Additionally, elevated PD-L1 levels and activated TGF-β signaling are observed in this context [38].

**Fig. 8:**
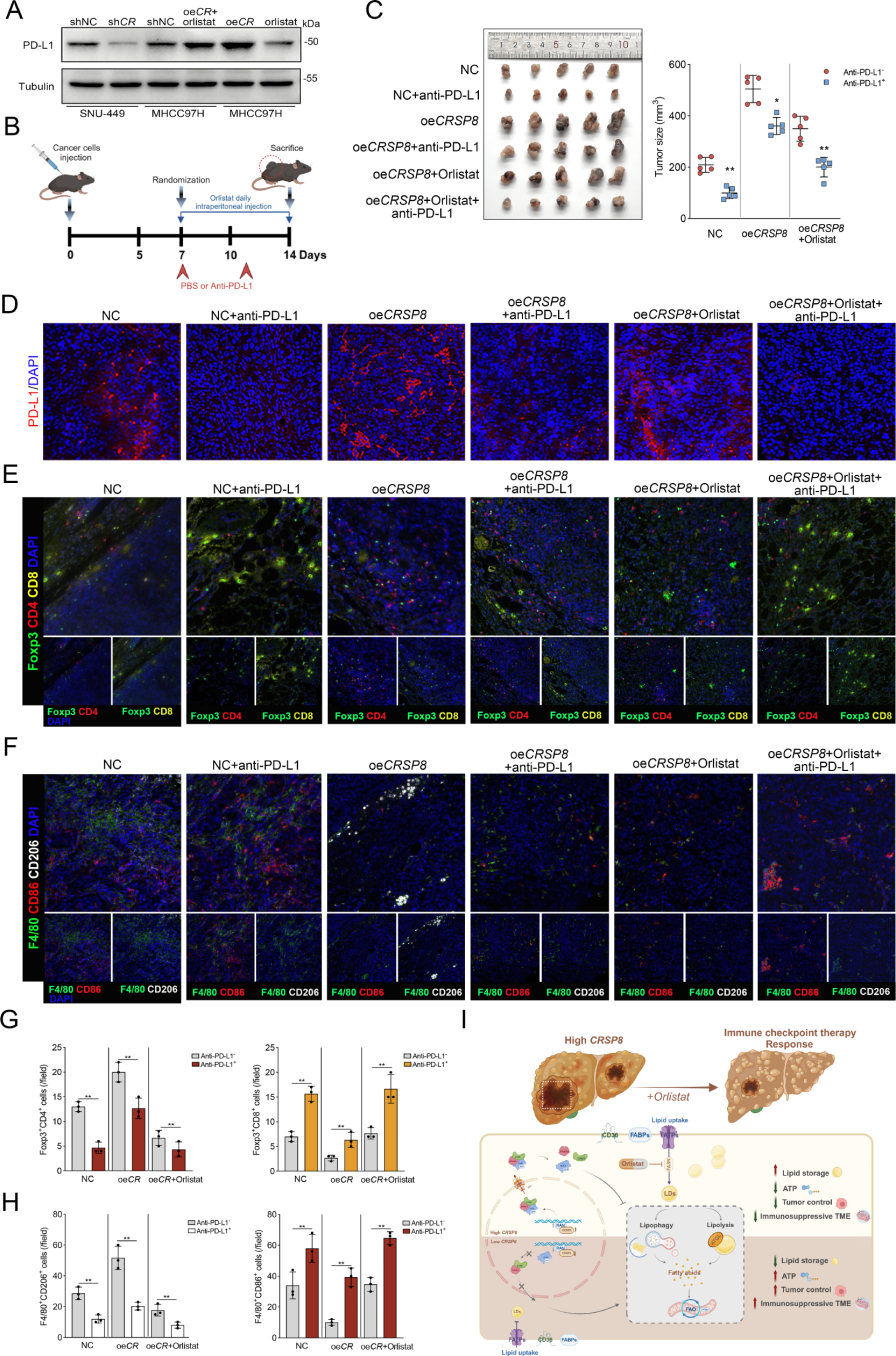
Interfering with fatty acid metabolism attenuates the immunosuppressive TME and response to anti-PD-L1 therapy of *CRSP8*-enriched HCC. (**A**) PD-L1 expression in cells with *CRSP8* knockdown/overexpression or orlistat treatment. (**B**) Schematic illustration of the establishment of the in vivo anti-PD-L1 therapy assay model. (**C**) *CRSP8* was overexpressed in Hepa1-6 cells, and a CDX model was established in C57BL/6 mice. The mice were either treated with orlistat or left untreated. The response of each group of mice to anti-PD-L1 was evaluated. (**D**-**F**) Immunofluorescence analysis of markers associated with the efficacy of anti-PD-L1 treatment in tumor tissues from the indicated groups of CDX models. Scale bar, 50 μm. (**G**, **H**) Fluorescence quantification of CD8^+^/CD4^+^Foxp3^+^ Treg cells and M1 or M2 macrophages in HCC tissues (*n* = 5). Data are presented as mean values ± SD. Differences between groups were determined using unpaired two-tailed Student’s *t*-test. *P < 0.05; **P < 0.01; ***P < 0.001. (**I**) A working model outlining the proposed mechanism. High *CRSP8* levels result in fatty acid reprograming by regulating the RAN/CRM1/PPARα nucleus–cytoplasm shuttling heterotrimer via transcriptionally activating *RAN*, resulting in decreased nuclear localization of PPARα and attenuated fatty acid oxidation, promoting HCC progression. Created with BioRender.com

Given that *CRSP8* plays a role in regulating FA metabolism, we investigated whether *CRSP8* modulates tumor progression and immune evasion in HCC. We found that *CRSP8* knockdown resulted in decreased PD-L1 expression, whereas overexpression reversed this effect. Notably, orlistat suppressed the increase in PD-L1 expression induced by *CRSP8* overexpression (Fig. 8A). In a subcutaneous tumor model, tumors with elevated *CRSP8* expression demonstrated a more favorable response to the combined treatment of orlistat and anti-PD-L1 therapy, compared to anti-PD-L1 therapy alone (Fig. 8B-C). Furthermore, immunofluorescence analysis indicated that orlistat treatment decreased PD-L1 levels (Fig. 8D) and restored the ratio of CD8(+) to CD4(+) Foxp3(+) regulatory T cells (Treg) in a cell-line-derived xenograft (CDX) model with *CRSP8* overexpression (Fig. 8E and G). We also observed increased M2 macrophage markers (F4/80 + CD206+) and decreased M1 macrophage markers (F4/80 + CD86+) in the orlistat-treated group (Fig. 8F and H). These findings suggest that orlistat enhances the responsiveness of CRSP8-enriched tumors to anti-PD-L1 therapy. We further found that in CRSP8-silenced cells, the protein levels of p-IKKα/β and p-IκBα were downregulated, while in CRSP8-overexpressing cells, they were upregulated. This indicates that CRSP8 activates the NF-κB signaling pathway in HCC cells, leading to the upregulation of PD-L1 expression and facilitating immune evasion (Fig. S9A). The mechanism by which high CRSP8 levels promote HCC progression is illustrated in Fig. 8I. Collectively, these results imply that targeting lipid metabolism in tumor cells may present a promising strategy to tackle the unfavorable TME in HCC.

### Combination of Orlistat and Sorafenib significantly inhibits HCC growth in vitro and in vivo

In addition to immune checkpoint inhibitor, we also focused on the clinically commonly used oral targeted drug for liver cancer, sorafenib, whose application is limited due to acquired resistance. Cell growth curve and colony formation assay were shown that sorafenib exhibits poor therapeutic efficacy in CRSP8-overexpressing HCC cells. However, we found that orlistat combined with sorafenib significantly inhibited HCC cell proliferation (Fig. S9B-C). In addition, Annexin V-propidium iodide (PI) assay was also performed to provide compelling evidence showing that orlistat in combination with sorafenib significantly increased the apoptosis rate in HepG2 cells with CRSP8 overexpression (Fig. S9D).

To further examine the sensitization effect of orlistat in vivo, we subcutaneously injected HepG2 cells with or without *CRSP8*-overexpressed into Balb/c nude mice, and randomly divided them into six groups receiving either vehicle control, sorafenib, or the combination treatment of orlistat and sorafenib every day. The results showed that, compared to sorafenib treated group, the combination treated group significantly suppressed tumor growth as indicated by smaller tumor volume and decreased tumor weight (Fig. S9E-F). The increased cell death and decreased Ki67 proliferation index further confirmed the synergistic effect of orlistat and sorafenib (Fig. S9G).

## Discussion

In this research, we discovered lipid metabolism genes related to HCC and chose CRSP8 for additional investigation. We explored the role and clinical relevance of CRSP8 in the context of HCC. The levels of CRSP8 expression were notably increased in HCC cohorts and were linked to poor clinical outcomes. Notably, we detected FA reprogramming in HCC cells with high *CRSP8* levels, potentially attributed to lipid accumulation caused by the overexpression of *CRSP8*. The tumorigenic role of *CRSP8* in melanoma and thyroid cancer were shown in previous studies [10, 11]. Nevertheless, the molecular functions of *CRSP8* in liver metabolism that led to tumorigenesis are still not well understood. This study represents the first comprehensive examination of the tumor-promoting role of CRSP8 in HCC, providing novel insights into its underlying mechanisms and highlighting CRSP8 as a potential therapeutic target for human HCC.

This research marks the first confirmation of elevated CRSP8 expression in HCC through extensive data analysis and validation with an independent internal cohort. Various cohorts have shown that elevated CRSP8 levels correlate with unfavorable prognosis. We analyzed CRSP8 mRNA and protein levels from various sources to ensure robust and dependable findings. Growing evidence indicates that obesity-related steatosis presents a significant public health challenge globally. Liver diseases are strongly associated with metabolic disorders [40]. Numerous enzymes involved in de novo FA synthesis, including *FASN*, *ACLY*, and *ACACA*, have been found to be significantly increased in various tumors, playing a role in the malignant characteristics of these tumors [41]. Moreover, earlier studies have confirmed the essential enzyme ACACA, associated with FA synthesis, is increased in various cancer tissues, and its elevated levels can significantly enhance cell proliferation [42]. Consistent with this, the present study showed that CRSP8 enhanced FA synthesis, thereby promoting HCC cell growth by upregulating the expression of ACACA. We also noted that elevated CRSP8 expression, when coupled with high levels of ACACA, was linked to a distinctly different histological pattern compared to cases with low CRSP8 expression and high ACACA levels.

The accumulation of excess lipids in hepatocytes, along with lipid overload (lipotoxicity), cell death cycles, regenerative growth, related inflammation and fibrosis, significantly contribute to the advancement of fatty liver disease to HCC [43]. Meanwhile, active mitochondria regulate the equilibrium between FAO and de novo lipogenesis, thereby affecting lipid levels and cell survival [44, 45]. It has been documented that LAMP2A loss in mouse liver results in hepatic steatosis due to impaired lipophagy [46]. Considering the significance of LD biology in liver disease progression, it is essential to comprehend the mechanisms that regulate LD accumulation and turnover [47]. PPARα acts as a crucial modulator of lipid metabolism [48] and functions as a nuclear receptor that governs the genes expression responsible for peroxisomal and mitochondrial FAO enzymes. It has been shown that long-term transcriptional regulation of autophagy involves nuclear receptors such as PPARα and farnesoid X receptor [49]. Etomoxir and Wort were used to investigate the association between lipophagy and FAO within the context of CRSP8 signaling. Notably, it exhibited significant resistance to Wort, indicating FAO functions downstream of lipophagy. Based on these findings, we conducted cell and animal experiments to assess whether PPARα activation enhances lipophagy and FAO independently of the mTOR signaling sensing mechanism. We analyzed phospho-mTOR expression, discovering CRSP8 had no impact on its expression (Fig. 5H). Additionally, our findings demonstrated that CRSP8 promoted nuclear localization of PPARα in both cell and animal experiments. The regulation of PPARα gene is significantly affected by various physiological factors, encompassing fasting, aging, and hormonal effects [50]. Numerous drugs and endogenous proteins have been identified as participants in hepatic FA metabolism by modulating PPARα levels [51]. Beyond the regulation of its expression, PPARα activity is influenced by both natural and synthetic ligands, such as FAs and their derivatives [50], post-translational modifications like phosphorylation [52], as well as the recruitment of cofactors [53]. As a nuclear transcription factor, the functionality of PPARα relies on its localization within the nucleus, which is governed by a dynamic balance of shuttling between the cytoplasm and nucleus. The transport of PPARα between these compartments is dependent on ligands [54]. Moreover, the ubiquitin ligase MuRF1 modulates cardiac PPARα through promoting its nuclear export via mono-ubiquitination, while MEK1 directly interacts with cardiac PPARα to inhibit its activity and facilitate its export from the nucleus [55]. Nonetheless, the precise mechanisms governing the subcellular localization of PPARα in hepatocytes remain unknown, particularly in the context of CRSP8 intervention. Our results indicated that *CRSP8* knockdown resulted in a decrease in PPARα nuclear export while leaving its import unaffected. Furthermore, we noted a significant correlation between *CRSP8* and *RAN* across various HCC cohorts, and in vitro studies indicated that *CRSP8* influences RAN expression in both mRNA and protein levels. Consequently, it appears that CRSP8 may facilitate lipid accumulation and the progression of HCC by upregulating RAN expression, which was confirmed through rescue assays. Besides FA synthesis, transcriptomic and lipidomic analyses indicate that CRSP8 is involved in maintaining cholesterol homeostasis. And CRSP8 may contribute to this process by modulating the uptake, efflux, and esterification of cholesterol. The underlying mechanisms require further investigation. Therefore, *CRSP8* may directly activate the transcription of *RAN*, which in turn inhibits the RAN/CRM1 interaction. This leads to a reduced RAN-CRM1-PPARα export heterotrimer formation. Consequently, CRSP8 can facilitate the nuclear localization of PPARα and enhance its activity in regulating lipophagy and FAO.

Cancer cells modify metabolic pathways to satisfy the unusual requirements for growth and survival. These cells often experience intermittent hypoxia within an acidic microenvironment, which disrupts the metabolism of cancer cells in an oxygen-rich environment. Previous investigations have shown that cancer cells in an acidic setting (pH 6.5) favor glutamine reductive metabolism [56] and FAO to generate acetyl-CoA for the tricarboxylic acid cycle. That leads to a greater reliance on FAs for energy production, along with the reactive oxygen species generation decrease, both of which contribute to tumor growth [57]. However, autophagy in HCC is complex and contradictory. Under normal conditions, basal autophagy eliminates impaired mitochondria and aberrant cells, preserving genomic stability as a tumor suppressor. However, once a tumor has formed, dysregulated autophagy aids the survival of HCC cells in various stressful environments, ultimately facilitating tumor growth [58]. When nutrients are scarce, cancer cells possess the ability to endure extended periods of nutrient deprivation by utilizing self-cannibalistic macroautophagy to release free amino acids and FAs [59]. In HCC characterized by lipid accumulation, tumor cells often degrade LDs to supply FAs to cells in need [60]. Conversely, lipid metabolism activates key oncogenic signaling pathways and is regarded as crucial for the onset and progression of tumors [61]. While autophagy has been shown to facilitate tumor growth and progression during later stages [62], the precise role of lipophagy is still not well understood. Through investigating the transcriptional regulation of CRSP8, we discovered that CRSP8 can influence the RAN-CRM1-PPARα heterotrimer by modulating RAN expression at the transcriptional level. Additionally, *CRSP8* interacts with *RAN* and is involved in the fusion of phagophores and LDs to facilitate lipid degradation. Since lipid accumulation is known to worsen HCC, the metabolic transformation of FAs into lipids, driven by reduced lipophagy and FAO, partially accounts for the oncogenic effects of CRSP8. Furthermore, the overexpression of *CRSP8* inhibited PPARα-mediated lipophagy and FAO activity.

FA metabolism presents a possible approach for tumor treatment, although it does not work effectively for all patients [63–65]. The molecular mechanisms underlying the variability in responses to FA metabolism inhibition remain unknown; however, discovering biomarkers to tailor treatments based on this inhibition could be advantageous for patients with HCC. There has been minimal research regarding the focus on FA metabolism alongside anti-PD-1/PD-L1 therapy; however, increasing data suggests the strategy may help to surmount resistance to immune checkpoint blockade (ICB). The FA reprogramming affects the PD-L1 levels in tumor, which may impact how these cells respond to ICB [66]. Malignant cells take advantage of the elevated lipid levels in obese patients and modify the TME to inhibit CD8 + T cell functions while facilitating cancer progression. Obesity in various mouse tumor models changes the metabolic characteristics of cancer cells, enhancing their absorption and utilization of FAs, which leads to an immunosuppressive TME that hinders both recruitment and function of CD8 + T cells [67]. Additionally, blocking the metabolic changes caused by obesity in murine colorectal carcinoma tumors reinstates the functions of CD8 + tumor-infiltrating lymphocytes (TILs) and enhances antitumor immune responses [67]. From a mechanistic perspective, in breast cancer tumors associated with obesity, CD8 + T cells bind to leptin and PD-1, resulting in diminished effector functions via STAT3 activation. This process activates FAO while suppressing glycolysis [68]. The binding of PD-1 enhances FAO in T cells by increasing CPT1A levels, a crucial enzyme that serves an important function in FAO [69]. Additionally, obesity in mice, humans, and nonhuman subjects results in elevated PD-1 levels and exhaustion of CD8 + T cells [70]. These findings support the idea that CD8 + T cells transition from glycolysis to FAO as they reach a state of exhaustion, underscoring the importance of investigating targeted metabolic reprogramming to rejuvenate CD8 + T cells and reduce resistance to ICB. In non-obese cancer models, similar with those in obese settings, an excess of FAs in the TME has also been demonstrated to hinder the function of CD8 + T cell. Furthermore, CD8 + TILs are marked by the expression of CD36, which aids in the absorption of oxidized low-density lipoproteins, phospholipids, and long-chain FAs [71]. HCC cells that overexpress *CRSP8* demonstrate alterations in FA metabolism. Therefore, we are interested in exploring the potential link between CRSP8 and how HCC cells respond to ICB, as this could offer valuable insights for creating immunotherapeutic approaches or forecasting patient outcomes. Following the knockdown of *CRSP8*, PD-L1 was decreased in HCC cells, indicating a risk of suppression in those with elevated levels of *CRSP8*, which was confirmed through studies conducted in an animal model. We found that in HCC tissues of the CDX model, the *CRSP8*-overexpressing group expressed high PD-L1, CD206, and CD86, which are clearly identified immunosuppressive factors [72]. This animal model showed no notable infiltration of CD8 + T cells. Immunosuppressive cells, including Tregs, macrophages, and myeloid-derived suppressor cells, depend significantly on external fatty acids to maintain the elevated rate of FAO, similar to cancer cells [73, 74]. In this context, elevated levels of FAs in the TME weaken the activity CD8 + T cells while providing advantages to malignant and immunosuppressive cells. Although *CRSP8* overexpression increases PD-L1 levels, which could elicit a stronger response, the benefits of this alteration on the immune environment are probably inadequate to offset the oncogenic consequences of FA metabolic reprogramming. Consequently, HCC cells that express high levels of CRSP8 are likely to show faster progression and decreased responsiveness to anti-PD-L1 therapies. Our findings further indicate that decreasing the expression of CRSP8 may inhibit tumor growth by diminishing FA functionality and enhancing immune suppression, as the overexpression of CRSP8 is associated with increased PD-L1 levels.

The combination of anti-PD-1 therapy and enhanced FA catabolism works together to significantly decrease the size of melanoma tumors in mice while fostering the antitumor metabolic reprogramming of CD8 + T cells [75]. Importantly, the variations seen in ICB responses among different cancer types emphasize the necessity for a more comprehensive understanding of the mechanisms that lead to resistance. To ensure that the benefits outweigh the risks, it is necessary to select appropriate patients with HCC for lipid depletion therapy. HCC cells that overexpress *CRSP8* demonstrated a heightened reliance on FAs for their energy needs. Therefore, we proposed that this metabolic shift makes HCC cells with elevated CRSP8 levels susceptible to lipid deprivation, which could serve as a potential approach for precision therapy in HCC. Our findings indicate that cells exhibiting elevated *CRSP8* levels are more susceptible to lipid deprivation or treatment with orlistat, an FASN inhibitor [76]. Consistent with previous studies, our study showed that orlistat downregulated PD-L1 levels in HCC cells with *CRSP8* overexpression, which improved immune suppression to some extent. Our results also revealed a general suppressive impact of orlistat on the growth of HCC cells, indicating that orlistat inhibits FA metabolism. Moreover, orlistat improved the anti-PD-L1 treatment response of HCC cells with high *CRSP8* expression. We found that CRSP8 is significantly enriched in HCC, where PD-L1 expression is also notably elevated. However, the specific mechanisms through which CRSP8 promotes tumor immune evasion remain to be further elucidated. Previous research has demonstrated that CRSP8 indirectly enhances NF-κB activity in thyroid cancer via its interaction with the NF-κB signaling pathway [10]. NF-κB is a critical transcription factor that plays an essential role in immune responses and inflammation. Its activation can promote immune evasion and upregulate PD-L1 expression. Furthermore, our transcriptomic analysis indicated that the knockdown of *CRSP8* resulted in the downregulation of the NF-κB pathway. Consequently, we conducted Western blot experiments to investigate whether CRSP8 can regulate the NF-κB signaling pathway. In *CRSP8*-silenced cells, the protein levels of p-IKKα/β and p-IκBα were downregulated, whereas these proteins were upregulated in CRSP8-overexpressing cells. These findings suggest that *CRSP8* activates the NF-κB signaling pathway in HCC, leading to increased PD-L1 expression and facilitating immune evasion.

Additionally, we focused on sorafenib, a commonly used oral targeted therapy for HCC, whose clinical application is often limited due to acquired resistance. Studies have shown that lipid accumulation significantly increases in sorafenib-resistant HCC cells, and supplementation with TG in normal HCC cells can prevent sorafenib-induced cell death. Conversely, reducing TG synthesis in sorafenib-resistant HCC cells enhances their sensitivity to sorafenib [77]. Considering that the metabolism of cancer cells is closely related to drug resistance, new targets associated with lipid metabolism are needed to ameliorate resistance to sorafenib. Our research provides both in vivo and in vitro validation for CRSP8-enriched HCC, demonstrating that combined treatment with orlistat and sorafenib can enhance the therapeutic effects of sorafenib. As a FASN inhibitor, orlistat exhibits a synergistic anti-tumor effect with sorafenib at low doses. Clinically, orlistat is used as an anti-obesity medication, and our findings offer promising prospects for future clinical translation.

Therefore, we conclude that individuals with HCC and elevated CRSP8 expression may be ideal candidates for lipid deprivation treatment, and that combining inhibition of FA metabolism with ICB shows potential for these patients. However, there is insufficient clinical evidence to substantiate the conclusion currently, which represents a limitation of the research.

In conclusion, we present evidence highlighting the clinical importance of increased CRSP8 levels in HCC. The upregulation of CRSP8 contributes to the advancement of HCC by altering FA metabolism. We have deciphered a mechanism underlying the adverse effect of CRSP8, which involves facilitation of nuclear localization of PPARα via transcriptional regulation of RAN. CRSP8 levels could serve as a prognostic biomarker and may assist in the development of effective therapies for HCC. Targeting fatty acid energy sources in HCC cells might be an advantageous approach for treating patients with high CRSP8 expression, and this benefit could be further amplified when used alongside ICB.

## Materials and methods

### Differentially expressed gene analysis and correlation analysis for public datasets

A total of 781 LMAGs were obtained from the Kyoto Encyclopedia of Genes and Genomes (KEGG) and Reactome databases, and 314 of these were identified as prognostic genes for HCC, using univariable Cox regression analysis. Integrative analysis of human hepatic transcriptome signatures (HCC datasets, GEO accession number GSE14520 and TCGA-LIHC) was conducted to interrogate the gene signatures critically involved in lipid metabolism. Three hub genes (*CRSP8*, *PAFAH1B3*, *GPD1L*) exhibited a two-fold difference in expression levels between tumors and the corresponding adjacent normal tissue.

RNA-seq mRNA expression profiles were extracted from TCGA-LIHC, ICGC-LIRI, and CNHPP. In total, 89 GEO HCC datasets were examined utilizing the IHGA (Integrative HCC Gene Analysis) online tool available at https://www.hccdatasph.cn/app/ihga. In this tool, the expression of *CRSP8* was compared between designated groups. The RNA-seq count data was analyzed in R. Gene expression data of cell lines was obtained from LIMORE project (https://www.biosino.org/limore/Faq). Pearson correlation method in R was used to examine the correlation between *CRSP8* and *RAN* in both tumor tissues and cell lines.

### Patients and clinical specimens

Paraffin-embedded and fresh tissue samples of HCC were collected from the Third Affiliated Hospital of Sun Yat-sen University in Guangzhou, China. In Cohort 1, a total of 30 paired snap-frozen samples were collected from HCC patients undergoing curative resections performed between October 2022 and October 2023 to assess mRNA and protein levels of CRSP8. For SYSU cohort, paraffin-embedded tissues were gathered, with data on OS and recurrence times available. These patients received curative resections from January to December 2008 and were monitored until December 2013. The protocols were approved by Ethical Review Committees of the Third Affiliated Hospital of Sun Yat-sen University, and all patients provided written informed consent. The research was carried out in compliance with the Helsinki and Istanbul Declarations.

### Cell lines and treatments

Cells were maintained in DMEM or RPMI1640 (Gibco, CA, USA) medium enriched with fetal bovine serum (Gibco, CA, USA) in a 5% CO_2_ atmosphere at 37 °C. All cell lines were sourced from the Cell Bank of the Chinese Academy of Sciences and have been recently verified through STR profiling. No contamination with mycoplasma was found.

### Animal experiments

All animal experiments received approval from the Laboratory Animal Ethics Committee of the South China Agricultural University and were performed following established guidelines. C57BL/6J male mice and BALB/c nude male mice (3–5 weeks old) were acquired from Zhuhai Bestest Bio-Tech Co., Ltd., and randomly assigned to different groups before cell inoculation (5 mice per group). Following a one-week acclimatization period in the specific pathogen-free animal facility, 1 × 10^7^ HepG2 cells (suspended in 100µL serum-free DMEM and Matrigel) or 2 × 10^6^ Hepa1-6 cells were injected subcutaneously to establish CDXs. One week after tumor cell inoculation, C57BL/6J mice in the indicated groups received orlistat (240 mg/kg per mouse, i.p.) [76] or anti-PD-L1 antibody (3 mg/kg per mouse, i.p.). When tumor volume reached approximately 100 mm^3^, the mice were randomized into treatment groups, including vehicle control, sorafenib (30 mg/kg/day, orally), orlistat (240 mg/kg/day, i.p.) or the combination, 5 mice per group. Tumor growth was monitored every three days. The C57BL/6J mice were then euthanized on day 14. And BALB/c nude mice were euthanized on day 30 and subcutaneous tumors were excised and weighed. The tumor volume in the CDX models was determined by applying the formula below: volume (mm^3^) = (width^2^ × length)/2. The harvested tumors were fixed with 10% formaldehyde for subsequent IHC and immunofluorescence analyses with the specified antibodies and utilized for measuring intra-tumoral TG levels. We used ARRIVE1 checklist when writing the report [78].

### Lentivirus system

The lentiviral vector system uses the GV lentiviral system from GeneChem, which includes the GV208 lentiviral vector for overexpression and the GV112 lentiviral vector for knockdown, as well as the pHelper1.0 and pHelper1.0 auxiliary plasmids. First, the fragment to be overexpressed or the RNAi sequence for knockdown is inserted into the lentiviral vector, and then the vector is co-transfected with the two auxiliary plasmids into HEK293T cells. After transfection for 72 h, the virus is collected, concentrated, and purified. The multiplicity of infection during cell infection is set to 10, and after 72 h of infection, 2µM puromycin screening is performed to establish a stable cell line.

### Quantitative RT-PCR (qRT-PCR)

Total RNA was isolated from cultured cell lines using Trizol (Invitrogen). And the Reverse Transcription Master Kit (TAKARA) and Power SYBR Green PCR Master Mix (TAKARA) were used according to the instructions. GAPDH was used as a reference control. The primers used for PCR are detailed in the Supplementary materials.

### Cell counting Kit-8 (CCK-8) assay

Cells were seeded in 96-well plates (4000–6000 cells per well). Then CCK-8 solution (Dojindo Laboratories, Kumamoto) was added and incubation was continued for 1–4 h. The absorbance was finally measured using a microplate reader (SpectraMax 190, Molecular Devices, USA).

### Colony formation assay

A predetermined quantity of cells (500–1000 cells) was plated in a 6-well plate and incubated for 10–14 days. The colonies were then stained with 0.1% crystal violet dye. After enough washing by PBS, cells were photographed and the cell colonies that contained more than 50 cells were counted.

### EdU proliferation assay

Cell proliferation was evaluated through the EdU assay. A total of 5000 cells were seeded into 96-well plates and then treated with diluted EdU solution at 37℃ for 3 h, after which images were taken. The relative abundance of EdU-positive cells was calculated by determining the ratio of labeled cells to the total numbers of cells in each field of view.

### Migration and invasion assays

1 × 10^4^ Cells were placed in the upper chamber of a 24-well plate (8-mm pore size; Corning, Cambridge, USA), with or without Matrigel coating on the membrane (BD Biosciences, USA). After a period of 48–72 h, cells that migrated and invaded through the membrane were fixed and stained using 1% crystal violet (Beyotime Biotechnology). The cells were then examined, photographed, and counted for further analysis.

### Western blot

To obtain the total protein from the samples, the RIPA buffer with protease inhibitors was utilized to extract the protein. The protein concentration was then measured using the BCA Protein Concentration Kit to ensure that equal quantities of protein were loaded. The SDS-PAGE gels were run to separate the proteins, and then were transferred onto PVDF membranes. To further analyze the proteins, the PVDF membranes were blocked with QuickBlock™ Buffer for 15 min at room temperature. After blocking, membranes were incubated with primary antibodies. To enhance the detection of the proteins, secondary antibodies were then added. Finally, the visual representation of the proteins was obtained using the BeyoECL Plus Kit. This kit allows for the detection of the proteins through the detection of the chemiluminescent signal generated by the bound secondary antibodies. The resulting bands were analyzed to determine the expression levels of the target proteins.

### Triglycerides and neutral lipids measurements

5 × 10^5^ Cells were placed in the upper chamber of a 6-well plate. To quantitatively assess triglycerides in cells, a Triglyceride Assay Kit (ab65336, Abcam, Cambridge, UK) was utilized. BODIPY 493/503 probe (D3922, Invitrogen) was applied to stain neutral lipid droplets. Cells were plated onto coverslips to reach the desired confluence. After 24 h, plates were and incubated in darkness for 30 min with a 2µM BODIPY 493/503 solution. The coverslips were subsequently rinsed three times with PBS, fixed in 4% PFA, stained with DAPI, and images were captured under a confocal microscope (Leica DM14000B).

### Co-immunoprecipitation (Co-IP)

Cells were harvested and lysed in co-IP lysis buffer on ice. After being centrifuged, the resulting lysates were immunoprecipitated using IgG or specific antibodies overnight. And protein G beads (Santa Cruz) were added to the protein-antibody complex and incubated for six hours. Cells underwent lysis again and 1 mg proteins were immunoprecipitated with Flag/HA-beads overnight. The complexes were subsequently washed with co-IP lysis buffer for 10 min each at 4 °C and collected by centrifugation. Proteins were released from the beads by denaturing them in a 100 °C loading buffer on a heat block (Thermo Fisher Scientific). Protein interactions were analyzed via western blotting.

### Autophagic flux

Recombinant adenovirus mRFP-GFP-LC3 (Hanbio Biotechnology) was transfected into HCC cells. The assessment of autophagic flux was conducted using the LC3 fluorescence-fusion protein, which includes yellow (autophagosomes) and red (autolysosomes) puncta under a confocal microscope (Leica DM14000B).

### Transmission electron microscope (TEM) assay

The cell samples were collected and fixed with 3% glutaraldehyde. Images were acquired using a TEM (JEM-1230, JEOL, USA). Two types of autophagic vacuoles including autophagosomes (double-membrane, absence of ribosomes, cytosol-similar density) and autolysosomes (single-membrane, much lower luminal density, containing light or dense amorphous) were quantified.

### Fluorescent fatty acid pulse-chase assay

Cells were treated with 1µM BODIPY™ FL-C16 (Thermo Scientific, USA) for 12 h. Then, cells were rinsed with complete medium and incubated for 12 h to enable the metabolism of fluorescent fatty acids. Mitochondria were stained with 200nM MitoTracker (Thermo Scientific, USA) for 30 min prior to imaging.

### FAO quantification

The FAO rate in HCC cells was assessed by fatty acid oxidation assay kit (ab217602, Abcam) in accordance with the guidelines provided by the manufacturer. Approximately 6 × 10^4^ cells per well were plated into 96-well plates. Cells treated with 2.5µM carbonyl cyanide 4-(trifluoromethoxy) phenylhydrazone (FCCP) were used as positive control, and cells treated with 40µM Etomoxir were used as negative control. Rates of FAO were calculated using slopes (m) from the linear portion of each profile as follows: FAO = m_untreated_– m_Etomoxir_.

### HIS-SIM imaging

Super-resolution imaging of mitochondria and palmitate structures was performed using commercialized HIS-SIM (High Intelligent and Sensitive SIM) provided by Guangzhou CSR Biotech Co. Ltd. Images were acquired using a 100×/1.5 NA oil immersion objective (Olympus). Cells were seeded in 8-well chambered cover glass or 6-well plate and maintained in a humidified chamber for live SIM imaging. SIM images were gathered and examined following the methods outlined earlier [79]. Sparse deconvolution was performed to improve the image quality [80].

### ATP production assay

5 × 10^5^ Cells were placed in the upper chamber of a 6-well plate. Then the ATP production in HCC cells was assessed by firefly luciferase-based ATP assay kit (Beyotime, S0026) in accordance with the guidelines provided by the manufacturer. Briefly, cells or tissues were fully lysed and centrifuged at 4℃, 12,000×g for 5 min. The supernatants (20 µl) were mixed with 100µL of ATP detection working dilution in Eppendorf tubes. Luminescence was measured by a luminometer.

### Immunofluorescence

Fresh tissue samples were fixed using 4% paraformaldehyde. Then samples were treated with 0.5% triton X-100. Paraffin sections are first dewaxed and then subjected to antigen repair. Primary antibodies incubation (CRSP8, Ki67, RAN, PPARα, CD86, CD206, Foxp3, F4/80, CD4, CD8, PD-L1) was performed at 4 °C at least 12 h and secondary antibodies conjugated with fluorescent dye were applied at room temperature for a duration of 1 h. Co-staining of several indexes was performed using a Four color mIHC Fluorescence kit (Hunan Aifang Biotechnology Co., Ltd., Changsha, China) based on the tyramide signal amplification (TSA) technology. Images were captured with a laser-scanning confocal microscope.

### Immunohistochemical staining (IHC)

The protocol for immunohistochemical staining of paraffin sections includes the following steps: deparaffinization in xylene, rehydration in a gradient of ethanol, H_2_O_2_ treatment, antigen retrieval, blocking with normal serum, incubation with the primary antibody (CRSP8), incubation with biotinylated secondary antibody, SAB complex incubation, DAB staining, and observation under a microscope. PBS should be used for washing after each step, and PBS should also be used to terminate the DAB staining.

### Hematoxylin and Eosin (H&E)

Tissues were fixed in 4% paraformaldehyde. Following dehydration, clearing, and paraffin embedding, tissue sections were then soaked in hematoxylin before being rinsed. Subsequently, they were stained with eosin and underwent a clearing process. Ultimately, neutral mounting medium was used to preserve the sections for observation and imaging.

### Luciferase reporter assay

We commissioned Hanbio Biotechnology Co., Ltd. to construct the dual luciferase reporter assay. The *RAN* promoter was subcloned between HindІІІ and KpnI sites of the pGL3 vector to construct a reporter plasmid. The Renilla luciferase control reporter vector pRL-TK served as a control. Cells were transfected with either *CRSP8*-specific siRNAs or overexpression plasmids. After 24 h, RAN promoter-driven luciferase plasmids were co-transfected into these cells along with renilla luciferase vectors. Following another 24-hour incubation post-transfection, cells were analyzed using the Dual-Luciferase^®^ Reporter Assay System (Promega, Madison, WI).

### Apoptosis assay

Apoptosis was measured based FACS analysis by using FITC-AV/PI staining. Cells with indicated treatment were collected, washed twice with cold PBS, resuspended with cold binding buffer, and subsequently stained with Annexin V-FITC and Propidium Iodide. Stained cells were then analyzed by using FACS Accuri C6.

### Extracellular flux analysis

Oxygen consumption rate (OCR) was measured using a Seahorse XFe96 Extracellular Flux Analyzer (Agilent Technologies). Cells were plated on Cell-Tak (Fisher)-coated XF 96-well plates at 2 × 10^4^ cells per well in assay medium (DMEM supplemented with 10mM glucose, 2mM L-glutamine and 1mM sodium pyruvate). Analyses of the OCR were performed at basal level, after subsequent injections of oligomycin (1µM), FCCP (1µM) and rotenone/antimycin mix (0.5µM).

### Molecular Docking

Docking analysis was performed using the Swiss-Dock software provided by the Swiss Institute of Bioinformatics (http://www.swissdock.ch/) [81]. The protein structure of RAN (UniProt: P62826) was selected as the target in Swiss-Dock, while the structure of PPARα (UniProt: Q07869) was uploaded as ligand molecular. And then the interactions among PPARα, RAN and CRM1 (UniProt: Q86 × 55) three molecules in sequence were analyzed. The interaction of residues among PPARα, RAN, and CRM1 was analyzed using PyMOL software (version 2.5.2).

### RNA sequencing (RNA-seq)

Total RNA was extracted using Trizol reagent (Invitrogen), with purity and concentration assessed by a NanoDrop 2000 spectrophotometer (Thermo Scientific, USA). Library preparation and sequencing were performed using the Illumina HiSeq X Ten platform. The FPKM values of genes were calculated using Cufflinks, while read counts were obtained through HTSeq-count. Differential expression analysis was carried out utilizing the DESeq (2012) R package. Gene Ontology (GO) term and KEGG pathway enrichment analysis of differentially expressed genes (DEGs) were performed using R. The transcriptome sequencing and analysis were conducted by KC Biotech (Wuhan, China).

### Metabolomic profiling

A combination of gas chromatography-time-of-flight mass spectrometry (GC-TOF/MS, LECO Corp., St Joseph, MI, USA) was used to quantify small-molecule metabolites in the indicated cells. The metabolites were recognized by comparing them to an internal library created with standard reference chemicals.

### Statistical analysis

SPSS 23.0 (IBM Corporation), R 4.1.0 software, and GraphPad Prism 7.0 (GraphPad Software, CA, USA) were used for statistical analyses. Data are expressed as mean ± standard deviation. The *t*-test was employed to assess differences between two groups. For RNA-seq or microarray data comparisons, Wilcoxon test was utilized. Correlations were analyzed using either Pearson’s correlation analysis. Survival data were examined with the Kaplan–Meier method. Categorical variables were assessed using the chi-square test. The R function cox.zph was utilized to assess the proportionality assumption. *P* < 0.05 was deemed to signify statistical significance.

## Electronic supplementary material

Below is the link to the electronic supplementary material.

Supplementary Material 1

Supplementary Material 2

Supplementary Material 3

Supplementary Material 4

## Data Availability

RNA-seq data of HCC cohorts from TCGA-LIHC were obtained in the UCSC Xena Public Data Hub (http://xena.ucsc.edu/public/). Data from the International Cancer Genome Consortium (ICGC-LIRI-JP) project were sourced from UCSC Xena Public Data Hub. Additionally, RNA-seq data for a Chinese HCC cohort was retrieved from CNHPP Data Portal (http://cnhpp.ncpsb.org.cn/). The online tool IHGA (https://www.hccdatasph.cn/app/ihga) was used to analyze microarray or RNA-seq data of 89 HCC cohorts. The datasets generated in this research can be requested from the corresponding authors. Furthermore, all other data supporting this study’s findings are accessible within the article and Supplementary materials, as well as available upon reasonable request from the corresponding author.

## Abbreviations

HCC: Hepatocellular carcinoma
PPARα: Peroxisome proliferator-activated receptorα
CRM1: Chromosome maintenance region 1
TME: Tumor microenvironment
ICB: Immune checkpoint blockade
FA: Fatty acid
FAO: Fatty acid oxidation
LD: Lipid droplet
TG: Triglyceride
LC3: Light chain 3
OS: Overall survival
RFS: Recurrence-free survival
RNA-Seq: RNA-sequencing
H&E: Hematoxylin and eosin
CDX: Cell line-derived xenografts
LMAG: Lipid metabolism associated gene
qRT-PCR: Quantitative reverse-transcription PCR
TCGA: The Cancer Genome Atlas
ACACA: Acetyl CoA carboxylase-1
CCK8: Cell Counting Kit-8
EMT: Epithelial-to-mesenchymal transition
GSEA: Gene set enrichment analysis
Wort: Wortmannin
CQ: Chloroquine
OCR: Oxygen consumption rate
IPZ: Importazole
LMB: Leptomycin B
co-IP: Co-immunoprecipitation
CPT1A: Carnitine palmitoyl transferase 1 A
TEM: Transmission electron microscopy
IHC: Immunohistochemistry
KEGG: Kyoto Encyclopedia of Genes and Genomes
IHGA: Integrative HCC Gene Analysis

## References

[1] Li Q, Pan X, Zhu D, et al. Circular RNA MAT2B promotes Glycolysis and malignancy of hepatocellular carcinoma through the miR-338-3p/PKM2 axis under hypoxic stress. Hepatology. 2019;70(4):1298–316. 31004447 10.1002/hep.30671

[2] Hu W, Zheng S, Guo H, et al. PLAGL2-EGFR-HIF-1/2α signaling loop promotes HCC progression and erlotinib insensitivity. Hepatology. 2021;73(2):674–91. 32335942 10.1002/hep.31293

[3] Hanahan D. Hallmarks of cancer: new dimensions. Cancer Discov. 2022;12(1):31–46. 35022204 10.1158/2159-8290.CD-21-1059

[4] Currie E, Schulze A, Zechner R, et al. Cellular fatty acid metabolism and cancer. Cell Metab. 2013;18(2):153–61. 23791484 10.1016/j.cmet.2013.05.017 PMC3742569

[5] Ansari SA, Morse RH. Mechanisms of mediator complex action in transcriptional activation. Cell Mol Life Sci. 2013;70(15):2743–56. 23361037 10.1007/s00018-013-1265-9 PMC11113466

[6] Gonzalez D, Hamidi N, Del Sol R, et al. Suppression of mediator is regulated by Cdk8-dependent Grr1 turnover of the Med3 coactivator. Proc Natl Acad Sci U S A. 2014;111(7):2500–5. 24550274 10.1073/pnas.1307525111 PMC3932902

[7] Ouimet M, Franklin V, Mak E, et al. Autophagy regulates cholesterol efflux from macrophage foam cells via lysosomal acid lipase. Cell Metab. 2011;13(6):655–67. 21641547 10.1016/j.cmet.2011.03.023 PMC3257518

[8] Cingolani F, Czaja MJ. Regulation and functions of autophagic lipolysis. Trends Endocrinol Metab. 2016;27(10):696–705. 27365163 10.1016/j.tem.2016.06.003 PMC5035575

[9] Wu W-Y, Kim H, Zhang C-L, et al. Clinical significance of autophagic protein LC3 levels and its correlation with XIAP expression in hepatocellular carcinoma. Med Oncol. 2014;31(8):108. 25005847 10.1007/s12032-014-0108-3

[10] Liao Y, Hua Y, Li Y, et al. CRSP8 promotes thyroid cancer progression by antagonizing IKKα-induced cell differentiation. Cell Death Differ. 2021;28(4):1347–63. 33162555 10.1038/s41418-020-00656-0 PMC8027816

[11] Tang R, Xu X, Yang W, et al. MED27 promotes melanoma growth by targeting AKT/MAPK and NF-κB/iNOS signaling pathways. Cancer Lett. 2016;373(1):77–87. 26797421 10.1016/j.canlet.2016.01.005

[12] He H, Dai J, Yang X, et al. Silencing of MED27 inhibits adrenal cortical carcinogenesis by targeting the Wnt/β-catenin signaling pathway and the epithelial-mesenchymal transition process. Biol Chem. 2018;399(6):593–602. 29730647 10.1515/hsz-2017-0304

[13] Che L, Chi W, Qiao Y, et al. Cholesterol biosynthesis supports the growth of hepatocarcinoma lesions depleted of fatty acid synthase in mice and humans. Gut. 2020;69(1):177–86. 30954949 10.1136/gutjnl-2018-317581 PMC6943247

[14] Broutier L, Mastrogiovanni G, Verstegen MM, et al. Human primary liver cancer-derived organoid cultures for disease modeling and drug screening. Nat Med. 2017;23(12):1424–35. 29131160 10.1038/nm.4438 PMC5722201

[15] Calvisi DF, Wang C, Ho C, et al. Increased lipogenesis, induced by AKT-mTORC1-RPS6 signaling, promotes development of human hepatocellular carcinoma. Gastroenterology. 2011;140(3):1071–83. 21147110 10.1053/j.gastro.2010.12.006 PMC3057329

[16] Yahagi N, Shimano H, Hasegawa K, et al. Co-ordinate activation of lipogenic enzymes in hepatocellular carcinoma. Eur J Cancer. 2005;41(9):1316–22. 15869874 10.1016/j.ejca.2004.12.037

[17] Svensson RU, Parker SJ, Eichner LJ, et al. Inhibition of acetyl-CoA carboxylase suppresses fatty acid synthesis and tumor growth of non-small-cell lung cancer in preclinical models. Nat Med. 2016;22(10):1108–19. 27643638 10.1038/nm.4181 PMC5053891

[18] Lally JSV, Ghoshal S, DePeralta DK et al. Inhibition of acetyl-CoA carboxylase by phosphorylation or the inhibitor ND-654 suppresses lipogenesis and hepatocellular carcinoma. Cell Metab. 2019;29(1). 10.1016/j.cmet.2018.08.020 PMC6643297 30244972

[19] Huber MA, Kraut N, Beug H. Molecular requirements for epithelial-mesenchymal transition during tumor progression. Curr Opin Cell Biol. 2005;17(5):548–58. 16098727 10.1016/j.ceb.2005.08.001

[20] Thiery JP, Acloque H, Huang RYJ, et al. Epithelial-mesenchymal transitions in development and disease. Cell. 2009;139(5):871–90. 19945376 10.1016/j.cell.2009.11.007

[21] Iershov A, Nemazanyy I, Alkhoury C, et al. The class 3 PI3K coordinates autophagy and mitochondrial lipid catabolism by controlling nuclear receptor PPARα. Nat Commun. 2019;10(1):1566. 30952952 10.1038/s41467-019-09598-9 PMC6451001

[22] Mana MD, Hussey AM, Tzouanas CN, et al. High-fat diet-activated fatty acid oxidation mediates intestinal stemness and tumorigenicity. Cell Rep. 2021;35(10):109212. 34107251 10.1016/j.celrep.2021.109212 PMC8258630

[23] Wickramasinghe NM, Sachs D, Shewale B et al. PPARdelta activation induces metabolic and contractile maturation of human pluripotent stem cell-derived cardiomyocytes. Cell Stem Cell. 2022;29(4). 10.1016/j.stem.2022.02.011 PMC11072853 35325615

[24] Olzmann JA, Carvalho P. Dynamics and functions of lipid droplets. Nat Rev Mol Cell Biol. 2019;20(3):137–55. 30523332 10.1038/s41580-018-0085-z PMC6746329

[25] Singh R, Kaushik S, Wang Y, et al. Autophagy regulates lipid metabolism. Nature. 2009;458(7242):1131–5. 19339967 10.1038/nature07976 PMC2676208

[26] Thumser AE, Storch J. Characterization of a BODIPY-labeled fluorescent fatty acid analogue. Binding to fatty acid-binding proteins, intracellular localization, and metabolism. Mol Cell Biochem. 2007;299(1–2):67–73. 16645726 10.1007/s11010-005-9041-2

[27] Nguyen TB, Louie SM, Daniele JR et al. DGAT1-dependent lipid droplet biogenesis protects mitochondrial function during starvation-induced autophagy. Dev Cell. 2017;42(1). 10.1016/j.devcel.2017.06.003 PMC5553613 28697336

[28] Pernas L, Bean C, Boothroyd JC et al. Mitochondria restrict growth of the intracellular parasite *Toxoplasma gondii* by limiting its uptake of fatty acids. Cell Metab. 2018;27(4). 10.1016/j.cmet.2018.02.018 29617646

[29] Rambold AS, Cohen S, Lippincott-Schwartz J. Fatty acid trafficking in starved cells: regulation by lipid droplet lipolysis, autophagy, and mitochondrial fusion dynamics. Dev Cell. 2015;32(6):678–92. 25752962 10.1016/j.devcel.2015.01.029 PMC4375018

[30] Yu SB, Pekkurnaz G. Mechanisms orchestrating mitochondrial dynamics for energy homeostasis. J Mol Biol. 2018;430(21):3922–41. 30089235 10.1016/j.jmb.2018.07.027 PMC6186503

[31] Giacomello M, Pyakurel A, Glytsou C, et al. The cell biology of mitochondrial membrane dynamics. Nat Rev Mol Cell Biol. 2020;21(4):204–24. 32071438 10.1038/s41580-020-0210-7

[32] Sekimoto T, Yoneda Y. Intrinsic and extrinsic negative regulators of nuclear protein transport processes. Genes Cells. 2012;17(7):525–35. 22672474 10.1111/j.1365-2443.2012.01609.x PMC3444693

[33] Umemoto T, Fujiki Y. Ligand-dependent nucleo-cytoplasmic shuttling of peroxisome proliferator-activated receptors, PPARα and PPARγ. Genes Cells. 2012;17(7):576–96. 22646292 10.1111/j.1365-2443.2012.01607.x

[34] Kehlenbach RH, Dickmanns A, Gerace L. Nucleocytoplasmic shuttling factors including Ran and CRM1 mediate nuclear export of NFAT in vitro. J Cell Biol. 1998;141(4):863–74. 9585406 10.1083/jcb.141.4.863 PMC2132762

[35] Stade K, Ford CS, Guthrie C, et al. Exportin 1 (Crm1p) is an essential nuclear export factor. Cell. 1997;90(6):1041–50. 9323132 10.1016/s0092-8674(00)80370-0

[36] Zhong J, He X, Gao X, et al. Hyodeoxycholic acid ameliorates nonalcoholic fatty liver disease by inhibiting RAN-mediated PPARα nucleus-cytoplasm shuttling. Nat Commun. 2023;14(1):5451. 37673856 10.1038/s41467-023-41061-8 PMC10482907

[37] Hu X, Yasuda T, Yasuda-Yosihara N, et al. Downregulation of 15-PGDH enhances MASH-HCC development via fatty acid-induced T-cell exhaustion. JHEP Rep. 2023;5(12):100892. 37942226 10.1016/j.jhepr.2023.100892 PMC10628853

[38] Murai H, Kodama T, Maesaka K, et al. Multiomics identifies the link between intratumor steatosis and the exhausted tumor immune microenvironment in hepatocellular carcinoma. Hepatology. 2023;77(1):77–91. 35567547 10.1002/hep.32573 PMC9970024

[39] Wang Y, Chen W, Qiao S et al. Lipid droplet accumulation mediates macrophage survival and Treg recruitment via the CCL20/CCR6 axis in human hepatocellular carcinoma. Cell Mol Immunol. 2024. 10.1038/s41423-024-01199-x PMC11443046 38942796

[40] Gluchowski NL, Becuwe M, Walther TC, et al. Lipid droplets and liver disease: from basic biology to clinical implications. Nat Rev Gastroenterol Hepatol. 2017;14(6):343–55. 28428634 10.1038/nrgastro.2017.32 PMC6319657

[41] Khwairakpam AD, Banik K, Girisa S, et al. The vital role of ATP citrate lyase in chronic diseases. J Mol Med (Berl). 2020;98(1):71–95. 31858156 10.1007/s00109-019-01863-0

[42] Rios Garcia M, Steinbauer B, Srivastava K et al. Acetyl-CoA carboxylase 1-dependent protein acetylation controls breast cancer metastasis and recurrence. Cell Metab. 2017;26(6). 10.1016/j.cmet.2017.09.018 29056512

[43] Font-Burgada J, Sun B, Karin M. Obesity and cancer: the oil that feeds the flame. Cell Metab. 2016;23(1):48–62. 26771116 10.1016/j.cmet.2015.12.015

[44] Schafer ZT, Grassian AR, Song L, et al. Antioxidant and oncogene rescue of metabolic defects caused by loss of matrix attachment. Nature. 2009;461(7260):109–13. 19693011 10.1038/nature08268 PMC2931797

[45] Svensson RU, Shaw RJ. Lipid synthesis is a metabolic liability of non-small cell lung cancer. Cold Spring Harb Symp Quant Biol. 2016;81. 10.1101/sqb.2016.81.030874 28062532

[46] Kaushik S, Cuervo AM. Degradation of lipid droplet-associated proteins by chaperone-mediated autophagy facilitates lipolysis. Nat Cell Biol. 2015;17(6):759–70. 25961502 10.1038/ncb3166 PMC4449813

[47] Mejhert N, Gabriel KR, Frendo-Cumbo S et al. The lipid droplet knowledge portal: A resource for systematic analyses of lipid droplet biology. Dev Cell. 2022;57(3). 10.1016/j.devcel.2022.01.003 PMC9129885 35134345

[48] Pawlak M, Lefebvre P, Staels B. Molecular mechanism of PPARα action and its impact on lipid metabolism, inflammation and fibrosis in non-alcoholic fatty liver disease. J Hepatol. 2015;62(3):720–33. 25450203 10.1016/j.jhep.2014.10.039

[49] Lee JM, Wagner M, Xiao R, et al. Nutrient-sensing nuclear receptors coordinate autophagy. Nature. 2014;516(7529):112–5. 25383539 10.1038/nature13961 PMC4267857

[50] Bougarne N, Weyers B, Desmet SJ, et al. Molecular actions of PPARα in lipid metabolism and inflammation. Endocr Rev. 2018;39(5):760–802. 30020428 10.1210/er.2018-00064

[51] Yang Z, Li P, Shang Q et al. CRISPR-mediated BMP9 ablation promotes liver steatosis via the down-regulation of PPARα expression. Sci Adv. 2020;6(48). 10.1126/sciadv.abc5022 PMC7695473 33246954

[52] Blanquart C, Barbier O, Fruchart J-C, et al. Peroxisome proliferator-activated receptor alpha (PPARalpha) turnover by the ubiquitin-proteasome system controls the ligand-induced expression level of its target genes. J Biol Chem. 2002;277(40):37254–9. 12118000 10.1074/jbc.M110598200

[53] Viswakarma N, Jia Y, Bai L et al. Coactivators in PPAR-regulated gene expression. PPAR Res. 2010;2010. 10.1155/2010/250126 PMC2929611 20814439

[54] Ziamajidi N, Khaghani S, Hassanzadeh G, et al. Amelioration by Chicory seed extract of diabetes- and oleic acid-induced non-alcoholic fatty liver disease (NAFLD)/non-alcoholic steatohepatitis (NASH) via modulation of PPARα and SREBP-1. Food Chem Toxicol. 2013;58:198–209. 23603006 10.1016/j.fct.2013.04.018

[55] Rodríguez JE, Liao J-Y, He J, et al. The ubiquitin ligase MuRF1 regulates PPARα activity in the heart by enhancing nuclear export via monoubiquitination. Mol Cell Endocrinol. 2015;413:36–48. 26116825 10.1016/j.mce.2015.06.008 PMC4523404

[56] Corbet C, Draoui N, Polet F, et al. The SIRT1/HIF2α axis drives reductive glutamine metabolism under chronic acidosis and alters tumor response to therapy. Cancer Res. 2014;74(19):5507–19. 25085245 10.1158/0008-5472.CAN-14-0705

[57] Corbet C, Pinto A, Martherus R, et al. Acidosis drives the reprogramming of fatty acid metabolism in cancer cells through changes in mitochondrial and histone acetylation. Cell Metab. 2016;24(2):311–23. 27508876 10.1016/j.cmet.2016.07.003

[58] Sun K, Guo XL, Zhao, Qd, et al. Paradoxical role of autophagy in the dysplastic and tumor-forming stages of hepatocarcinoma development in rats. Cell Death Dis. 2013;4(2):e501. 10.1038/cddis.2013.35 PMC3734842 23429287

[59] Boya P, Reggiori F, Codogno P. Emerging regulation and functions of autophagy. Nat Cell Biol. 2013;15(7):713–20. 23817233 10.1038/ncb2788 PMC7097732

[60] Dumas J-F, Brisson L, Chevalier S et al. Metabolic reprogramming in cancer cells, consequences on pH and tumour progression: integrated therapeutic perspectives with dietary lipids as adjuvant to anticancer treatment. Semin Cancer Biol. 2017;43. 10.1016/j.semcancer.2017.03.004 28323020

[61] Tennant DA, Durán RV, Gottlieb E. Targeting metabolic transformation for cancer therapy. Nat Rev Cancer. 2010;10(4):267–77. 20300106 10.1038/nrc2817

[62] Menendez JA, Lupu R. Fatty acid synthase and the lipogenic phenotype in cancer pathogenesis. Nat Rev Cancer. 2007;7(10):763–77. 17882277 10.1038/nrc2222

[63] Diana A, Wang LM, D’Costa Z, et al. Prognostic value, localization and correlation of PD-1/PD-L1, CD8 and FOXP3 with the desmoplastic stroma in pancreatic ductal adenocarcinoma. Oncotarget. 2016;7(27):40992–1004. 27329602 10.18632/oncotarget.10038 PMC5173037

[64] Shen T, Zhou L, Shen H, et al. Prognostic value of programmed cell death protein 1 expression on CD8 + T lymphocytes in pancreatic cancer. Sci Rep. 2017;7(1):7848. 28798308 10.1038/s41598-017-08479-9 PMC5552822

[65] Winograd R, Byrne KT, Evans RA, et al. Induction of T-cell immunity overcomes complete resistance to PD-1 and CTLA-4 Blockade and improves survival in pancreatic carcinoma. Cancer Immunol Res. 2015;3(4):399–411. 25678581 10.1158/2326-6066.CIR-14-0215 PMC4390506

[66] Laubach K, Turan T, Mathew R, et al. Tumor-intrinsic metabolic reprogramming and how it drives resistance to anti-PD-1/PD-L1 treatment. Cancer Drug Resist. 2023;6(3):611–41. 37842241 10.20517/cdr.2023.60 PMC10571065

[67] Ringel AE, Drijvers JM, Baker GJ et al. Obesity shapes metabolism in the tumor microenvironment to suppress anti-tumor immunity. Cell. 2020;183(7). 10.1016/j.cell.2020.11.009 PMC8064125 33301708

[68] Zhang C, Yue C, Herrmann A et al. STAT3 activation-induced fatty acid oxidation in CD8 + T effector cells is critical for obesity-promoted breast tumor growth. Cell Metab. 2020;31(1). 10.1016/j.cmet.2019.10.013 PMC6949402 31761565

[69] Patsoukis N, Bardhan K, Chatterjee P, et al. PD-1 alters T-cell metabolic reprogramming by inhibiting Glycolysis and promoting lipolysis and fatty acid oxidation. Nat Commun. 2015;6:6692. 25809635 10.1038/ncomms7692 PMC4389235

[70] Wang Z, Aguilar EG, Luna JI, et al. Paradoxical effects of obesity on T cell function during tumor progression and PD-1 checkpoint Blockade. Nat Med. 2019;25(1):141–51. 30420753 10.1038/s41591-018-0221-5 PMC6324991

[71] Xu S, Chaudhary O, Rodríguez-Morales P et al. Uptake of oxidized lipids by the scavenger receptor CD36 promotes lipid peroxidation and dysfunction in CD8 + T cells in tumors. Immunity. 2021;54(7). 10.1016/j.immuni.2021.05.003 PMC9273026 34102100

[72] Kubota K, Moriyama M, Furukawa S, et al. CD163 + CD204 + tumor-associated macrophages contribute to T cell regulation via interleukin-10 and PD-L1 production in oral squamous cell carcinoma. Sci Rep. 2017;7(1):1755. 28496107 10.1038/s41598-017-01661-z PMC5431876

[73] Huang SC-C, Everts B, Ivanova Y, et al. Cell-intrinsic lysosomal lipolysis is essential for alternative activation of macrophages. Nat Immunol. 2014;15(9):846–55. 25086775 10.1038/ni.2956 PMC4139419

[74] Wang H, Franco F, Tsui Y-C, et al. CD36-mediated metabolic adaptation supports regulatory T cell survival and function in tumors. Nat Immunol. 2020;21(3):298–308. 32066953 10.1038/s41590-019-0589-5 PMC7043937

[75] Zhang Y, Kurupati R, Liu L et al. Enhancing CD8 + T cell fatty acid catabolism within a metabolically challenging tumor microenvironment increases the efficacy of melanoma immunotherapy. Cancer Cell. 2017;32(3). 10.1016/j.ccell.2017.08.004 PMC5751418 28898698

[76] Kridel SJ, Axelrod F, Rozenkrantz N, et al. Orlistat is a novel inhibitor of fatty acid synthase with antitumor activity. Cancer Res. 2004;64(6):2070–5. 15026345 10.1158/0008-5472.can-03-3645

[77] Li Y, Yang W, Zheng Y, et al. Targeting fatty acid synthase modulates sensitivity of hepatocellular carcinoma to Sorafenib via ferroptosis. J Exp Clin Cancer Res. 2023;42(1):6. 36604718 10.1186/s13046-022-02567-z PMC9817350

[78] Kilkenny C, Browne WJ, Cuthill IC, et al. Improving bioscience research reporting: the ARRIVE guidelines for reporting animal research. PLoS Biol. 2010;8(6):e1000412. 20613859 10.1371/journal.pbio.1000412 PMC2893951

[79] Huang X, Fan J, Li L, et al. Fast, long-term, super-resolution imaging with Hessian structured illumination microscopy. Nat Biotechnol. 2018;36(5):451–9. 29644998 10.1038/nbt.4115

[80] Zhao W, Zhao S, Li L, et al. Sparse Deconvolution improves the resolution of live-cell super-resolution fluorescence microscopy. Nat Biotechnol. 2022;40(4):606–17. 34782739 10.1038/s41587-021-01092-2

[81] Grosdidier A, Zoete V, Michielin O. SwissDock, a protein-small molecule Docking web service based on EADock DSS. Nucleic Acids Res. 2011;39(Web Server issue):W270–7. 21624888 10.1093/nar/gkr366 PMC3125772

