# Supplementary material for: CRSP8-driven fatty acid metabolism reprogramming enhances hepatocellular carcinoma progression by inhibiting RAN-mediated PPARα nucleus-cytoplasm shuttling
Source: J Exp Clin Cancer Res. 2025 Mar 11;44:93. doi: 10.1186/s13046-025-03329-3 (PMC11895297; doi:10.1186/s13046-025-03329-3)
Supplement: Supplementary file 3 — Supplementary Material 3 [file 13046_2025_3329_MOESM3_ESM.doc]

**Supplementary Movie Legends**

**Movie S1.** Dynamics of fatty acids and mitochondria in SNU-449 cells.

**Movie S2.** Dynamics of fatty acids and mitochondria in SNU-449 cells with *CRSP8* knockdown, related to Figure 4K.
