# Supplementary material for: CRSP8-driven fatty acid metabolism reprogramming enhances hepatocellular carcinoma progression by inhibiting RAN-mediated PPARα nucleus-cytoplasm shuttling
Source: J Exp Clin Cancer Res. 2025 Mar 11;44:93. doi: 10.1186/s13046-025-03329-3 (PMC11895297; doi:10.1186/s13046-025-03329-3)
Supplement: Supplementary file 4 — Supplementary Material 4 [file 13046_2025_3329_MOESM4_ESM.pdf]

CRSP8-driven Fatty Acid Metabolism Reprogramming Enhances Hepatocellular Carcinoma Progression by Inhibiting RAN-mediated PPARα Nucleus-cytoplasm Shuttling

Yuxi Lin, Zhixing Liang, Zhiyan Weng, Xiaofang Liu, Feng Zhang, Yutian Chong

Supplementary Materials..2

Supplementary Figure and Figure legends 8

Fig. S1 8

Fig. S2 10

Fig. S3 12

Fig. S4 13

Fig. S5 15

Fig. S6 17

Fig. S7 18

Fig. S8 20

Fig. S9 22

Supplementary Materials

The specific primers used for qRT-PCR in the present study

**Gene**	**Forward primer**	**Reverse primer**
Human *CRSP8*	GTGTTCGACTGCCTGAAGGA	GGACAAGATTGTGGGCTGA
Human *PPARα*	TTCGCAATCCATCGGCGAG	CCACAGGATAAGTCACCGAGG
Human *PPARβ/δ*	CAGGGCTGACTGCAAACGA	CTGCCACAATGTCTCGATGTC
Human *PPARγ*	TACTGTCGGTTTCAGAAATGCC	GTCAGCGGACTCTGGATTCAG
Human *RAN*	GGTGGTACTGGAAAAACGACC	CCCAAGGTGGCTACATACTTCT
Human *ACACA*	CATGCGGTCTATCCGTAGGTG	GTGTGACCATGACAACGAATCT
Human *ACLY*	TCGGCCAAGGCAATTTCAGAG	CGAGCATACTTGAACCGATTCT
Human *CD36*	GGCTGTGACCGGAACTGTG	AGGTCTCCAACTGGCATTAGAA
Human *FASN*	AAGGACCTGTCTAGGTTTGATGC	TGGCTTCATAGGTGACTTCCA
Human *FABP5*	TGAAGGAGCTAGGAGTGGGAA	TGCACCATCTGTAAAGTTGCAG
Human *FABP4*	ACTGGGCCAGGAATTTGACG	CTCGTGGAAGTGACGCCTT
Human *SCD1*	TCTAGCTCCTATACCACCACCA	TCGTCTCCAACTTATCTCCTCC
Human *ACSL1*	CTTATGGGCTTCGGAGCTTTT	CAAGTAGTGCGGATCTTCGTG
Human *ACSL3*	GCCGAGTGGATGATAGCTGC	ATGGCTGGACCTCCTAGAGTG
Human *ACSL5*	CTCAACCCGTCTTACCTCTTCT	GCAGCAACTTGTTAGGTCATTG
Human *SREBP1*	ACAGTGACTTCCCTGGCCTAT	GCATGGACGGGTACATCTTCAA
Human *SREBP2*	AACGGTCATTCACCCAGGTC	GGCTGAAGAATAGGAGTTGCC
Human *HMGCR*	TGATTGACCTTTCCAGAGCAAG	CTAAAATTGCCATTCCACGAGC
Human *p62*	GCACCCCAATGTGATCTGC	CGCTACACAAGTCGTAGTCTGG
Human *LC3-I/II*	GATGTCCGACTTATTCGAGAGC	TTGAGCTGTAAGCGCCTTCTA
Human *ATG5*	AAAGATGTGCTTCGAGATGTGT	CACTTTGTCAGTTACCAACGTCA
Human *ATGL*	GGCTTCCTCGGCGTCTACTA	TTTACCAGGTTGAAGGAGGGG
Human *LAMP1*	TCTCAGTGAACTACGACACCA	AGTGTATGTCCTCTTCCAAAAGC
Human *PLIN2*	ATGGCATCCGTTGCAGTTGAT	GGACATGAGGTCATACGTGGAG
Human *ACOX1*	ACTCGCAGCCAGCGTTATG	AGGGTCAGCGATGCCAAAC
Human *CPT1A*	TCCAGTTGGCTTATCGTGGTG	TCCAGAGTCCGATTGATTTTTGC

Human *β-Tubulin*	GGCCAAGGGTCACTACACG	GCAGTCGCAGTTTTCACACTC

3 **Antibody used in the present study**

**Antibody**	**Supplier**	**Catalog number**
CRSP8	Invitrogen	PA5-101981/52374
CRSP8	Immunoway	YT1122
β-Tubulin	Cell Signaling Technology	2146
PCNA	Cell Signaling Technology	13110
Snail	Cell Signaling Technology	3879
E-cadherin	Cell Signaling Technology	3195
N-cadherin	Cell Signaling Technology	13116
β-catenin	Cell Signaling Technology	9562
FASN	HUABIO	ET1701-91
ACC1	HUABIO	ET1609-77
ACLY	Cell Signaling Technology	9441
CD36	Abcam	ab33625
FABP4	Cell Signaling Technology	2120
FABP5	Cell Signaling Technology	39926
ATGL	Cell Signaling Technology	2138
LAMP1	Abcam	ab25630
LAMP1	Cell Signaling Technology	9091
ATG5	Cell Signaling Technology	12994
PLIN2	Abcam	ab108323
p62	Cell Signaling Technology	39749
LC3	Cell Signaling Technology	4108
PPARα	Abcam	ab126285
PPARα	Abclonal	A24835
ACOX1	Proteintech	10957-1-Ig
CPT1A	Proteintech	15184-1-Ig
mTOR	Cell Signaling Technology	2983

Phospho-mTOR	Cell Signaling Technology	2971
Histone H3	Cell Signaling Technology	9715
PPARβ/δ	Cell Signaling Technology	74076
PPARγ	Cell Signaling Technology	2430
CRM1	Santa Cruz Biotechnology	sc-74454
CRM1	Proteintech	66763-1-Ig
RAN	Proteintech	67500-1-Ig
RAN	Santa Cruz Biotechnology	sc-271376
GAPDH	Cell Signaling Technology	2118S
Ki67	Cell Signaling Technology	9129
IKKα	Cell Signaling Technology	2682
IKKβ	Cell Signaling Technology	8943
Phospho-IKKα/β	Cell Signaling Technology	2697
IκBα	Cell Signaling Technology	9242
Phospho-IκBα	Cell Signaling Techonology	2859
p65	Cell Signaling Techonology	8242
Phospho-p65	Cell Signaling Techonology	3033
Flag	Sigma-Aldrich	F1804
HA	Proteintech	51064-2-AP
Mouse Control IgG Antibody	Abclonal	AC011
PD-L1	Proteintech	66248-1-Ig
CD86	Cell Signaling Technology	19589S
CD206	Affinity	DF4149
Foxp3	Servicebio	GB112325
CD8	Abclonal	A11856
CD4	Abcam	ab183685
F4/80	Cell Signaling Technology	30325
Anti-rabbit IgG, HRP-linked	Cell Signaling Technology	7076
Anti-mouse IgG, HRP-linked	Cell Signaling Technology	7074

Anti-Rabbit IgG, Alexa Fluor 488	Thermo Fisher Scientific	A21206
Anti-Rabbit IgG, Alexa Fluor 647	Thermo Fisher Scientific	A21222
Anti-Mouse IgG, Alexa Fluor 488	Thermo Fisher Scientific	A11001
Anti-Mouse IgG, Alexa Fluor 647	Thermo Fisher Scientific	A21235

The reagent used in the present study

**Reagent**	**Supplier**	**Catalog number**
Rapamycin	MedChemExpress	HY-10219
Chloroquine	Sigma-Aldrich	C6628
RIPA	Beyotime Biotechnology	P0013B
DAPI	Beyotime Biotechnology	C1005
Triton X-100	Beyotime Biotechnology	ST797-100ml
PMSF	Solarbio	P0100
TRIzol reagent	Invitrogen	15596018
Phosphatase Inhibitor Cocktail I	MedChemExpress	HY-k0021
Phosphatase Inhibitor Cocktail II	MedChemExpress	HY-k0022
Dulbecco's Modified Eagle Medium	Gibco	11995065
Roswell Park Memorial Institute 1640 medium	Gibco	C11875500BT
Fetal bovine serum	Gibco	10099-141
Penicillin-Streptomycin	Thermo Fisher Scientific	15140122
Matrigel	Corning	356234
BODIPY 493/503	Invitrogen	D3922
Lysotracker™ Green DND-26	Thermo Fisher Scientific	L7526
Mito Tracker™ Deep Red FM	Thermo Fisher Scientific	M22426
Phenylhydrazone (FCCP)	Sanbio	1528-10
Oligomycin	Sigma-Aldrich	75351
Antimycin A	Sigma-Aldrich	A8674
Etomoxir	MedChemExpress	HY-50202
BODIPY™ FL C16	Thermo Fisher Scientific	D3821

Orlistat	MedChemExpress	HY-B0218
QuickBlock™ blocking buffer	Beyotime Biotechnology	P0220
QuickBlock™ Primary Antibody Dilution Buffer	Beyotime Biotechnology	P0256
QuickBlock™ Secondary Antibody Dilution Buffer	Beyotime Biotechnology	P0258
Trypsin	Cytiva	SH30042.02
Crystal Violet Staining Solution	Beyotime Biotechnology	C0121
Leptomycin B	MedChemExpress	HY-16909
Importazole	MedChemExpress	HY-101091

Kits used in the present study

**Kits**	**Supplier**	**Catalog number**
ATP Assay kit	Beyotime Biotechnology	S0026
Dual-Luciferase Reporter Assay System	Promega	E1910
BCA Protein Concentration kit	Beyotime Biotechnology	P0012
Cell-Light EdU Apollo567 In Vitro kit	RiboBio Co., Ltd.	C10310-1
High-capacity cDNA Reverse Transcription Kit	TAKARA	RR037B
Co-immunoprecipitation Kit	Invitrogen	10007D
Tyramide Signal Amplification Kit	Afantibody	AFIHC024
SYBR Green Master Mix	TAKARA	RR420A
BeyoECL Plus kit	Beyotime Biotechnology	P0018M
Triglyceride Assay	Abcam	ab65336
Cholesterol Assay	Abcam	ab133116
Fatty Acid Oxidation Assay	Abcam	ab217602
Cell Counting Kit-8 test (CCK8)	Dojindo Laboratories, Kumamoto	CK04
Nuclear and Cytoplasmic Protein Extraction Kit	Beyotime Biotechnology	P0028
Annexin V-EGFP/PI Apoptosis Detection Kit	Keygentec	KGA1101

Supplementary Figure and Figure legends

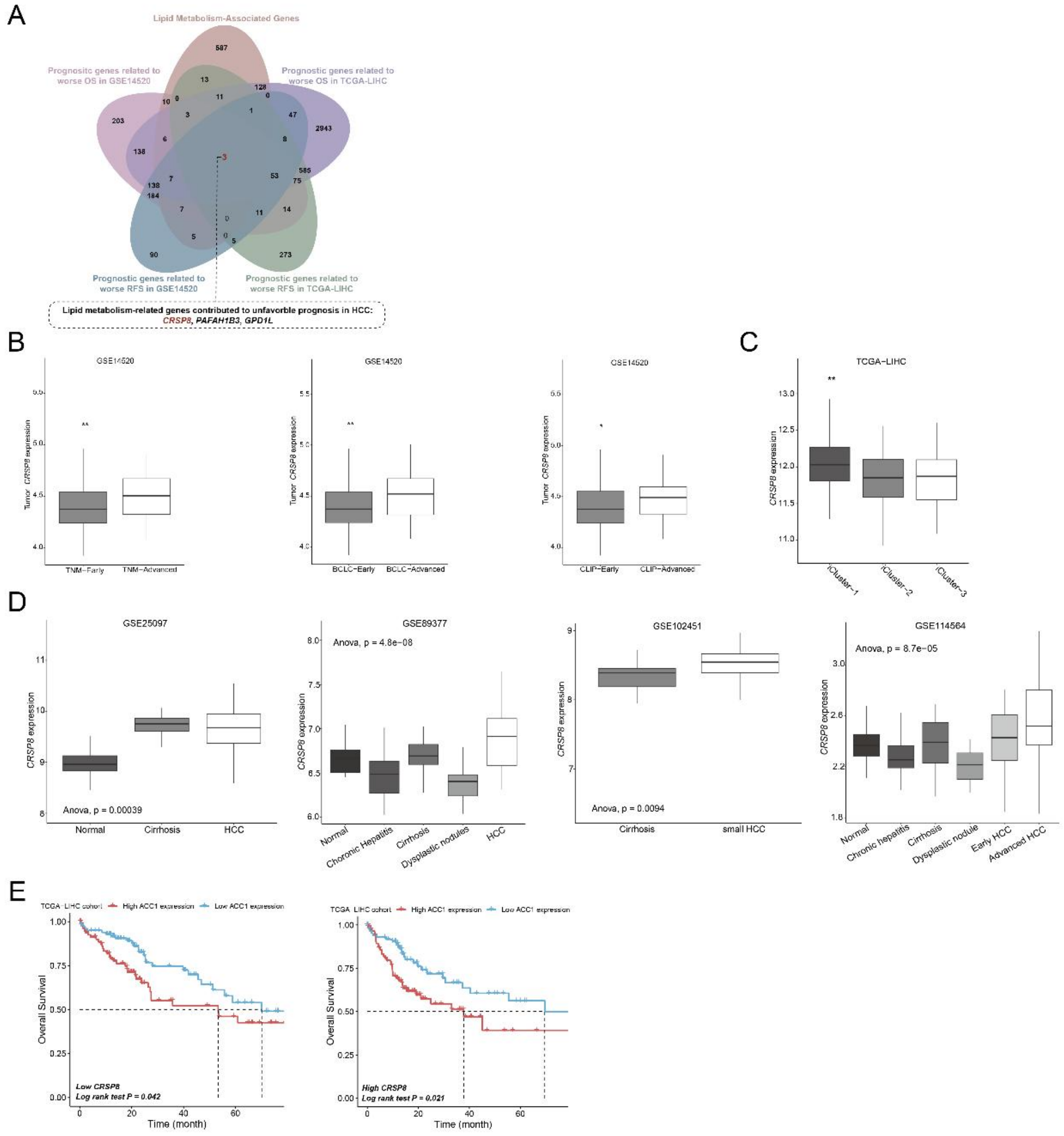

Fig. S1. The expression difference of *CRSP8* mRNA in different clinical stages, progression and molecular subtypes in HCC were analyzed using Integrative HCC Gene Analysis (IHGA) online tools. Data were obtained in indicated public HCC-related datasets. Plots were created by IHGA online tools.

(A) Lipid metabolism associated genes profiling strategy.

(B) In different clinical staging systems, patients were divided into late-stage and early-stage groups, and the *CRSP8* mRNA levels were compared between the two groups. Early= (TNM: I-II)/(BCLC:0/A-B)/(CLIP:<=2); Advanced= (TNM: III-IV)/(BCLC:C-D)/(CLIP:>2).

(C) The mRNA levels of *CRSP8* were compared in tumor tissues from HCC patients with different molecular subtypes.

(D) The levels of *CRSP8* mRNA were investigated at different step of HCC formation. Data are shown as median; *$P < 0.05$; **$P < 0.01$; ***$P < 0.001$.

(E) Overall HCC survival data obtained from the TCGA-LIHC database for tumors showing CRSP8^{high} expression (above the mean) versus CRSP8^{low} expression (below the mean) with either ACACA^{Low} or ACACA^{High} expression.

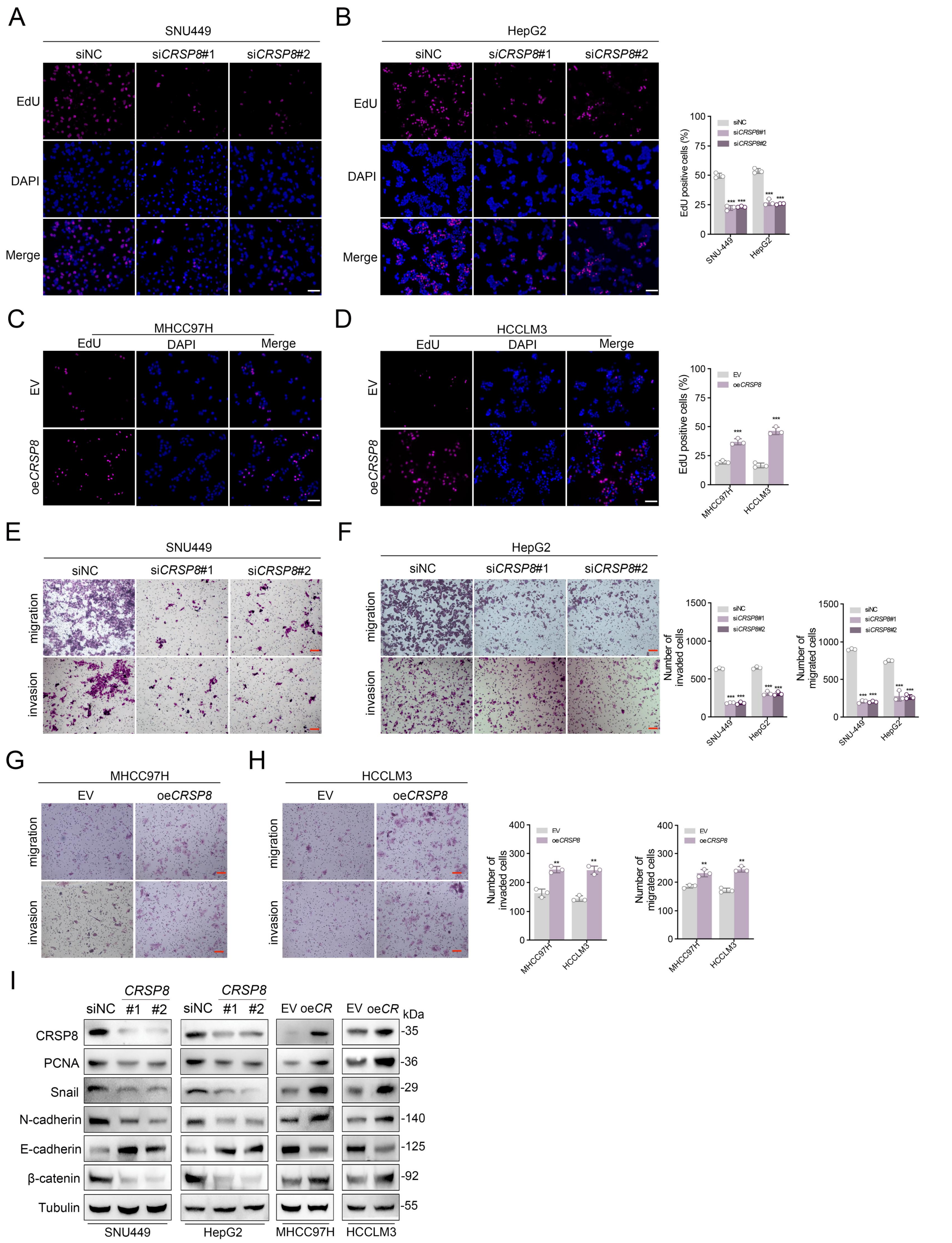

Fig. S2. *CRSP8* promotes the proliferation and migration of HCC cells *in vitro.*

(A-D) 5-ethynyl-20-deoxyuridine (EdU) assays of HCC cells treated as indicated, n = 3.

(E-H) Transwell migration and invasion assay using the indicated HCC cells.

(I) Western blot analysis of the expression of proliferation and EMT markers (PCNA, E-cadherin, N-cadherin, β-catenin, Snail) in HCC cells following *CRSP8* silencing or overexpression. Data are mean ± SD. Unpaired two-tailed student's *t*-test or One-way ANOVA for multiple comparisons

were used to analyze the results presented in A-D, respectively. *$P < 0.05$; **$P < 0.01$; ***$P < 0.001$.

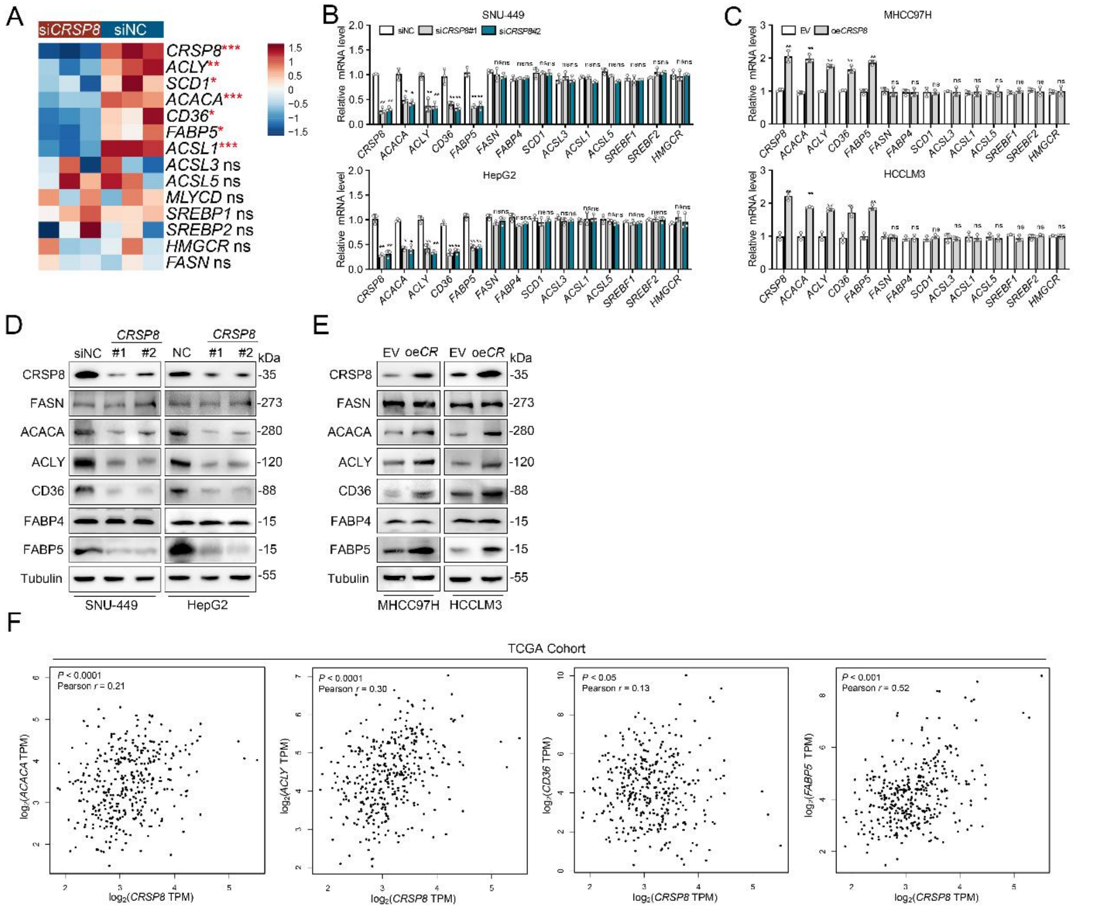

Fig. S3. ***CRSP8* promotes de novo lipogenesis via upregulation of lipogenic enzymes.**

(A) Heatmap showing dysregulation of genes involved in fatty acid synthesis (*SREBP1*, *ACLY, SCD1*, *ACACA*, *MLYCD*, *ACSL1*, *ACSL3*, *ACSL5*, and *FABP5*), fatty acid uptake (*CD36*), and cholesterol biosynthesis (*SREBP2* and *HMGCR*).

(B-C) Relative mRNA levels of indicated regulators of lipid metabolism following siRNA knockdown or plasmid overexpression of *CRSP8* in indicated HCC cells.

(D-E) Relative protein levels of indicated regulators of lipid metabolism following siRNA knockdown or plasmid overexpression of *CRSP8* in indicated HCC cells.

(F) Scatter plot analysis of correlation between mRNA levels of *CRSP8* and *ACACA*, *ACLY*, *CD36*, or *FABP5* in TCGA-LIHC cohort.

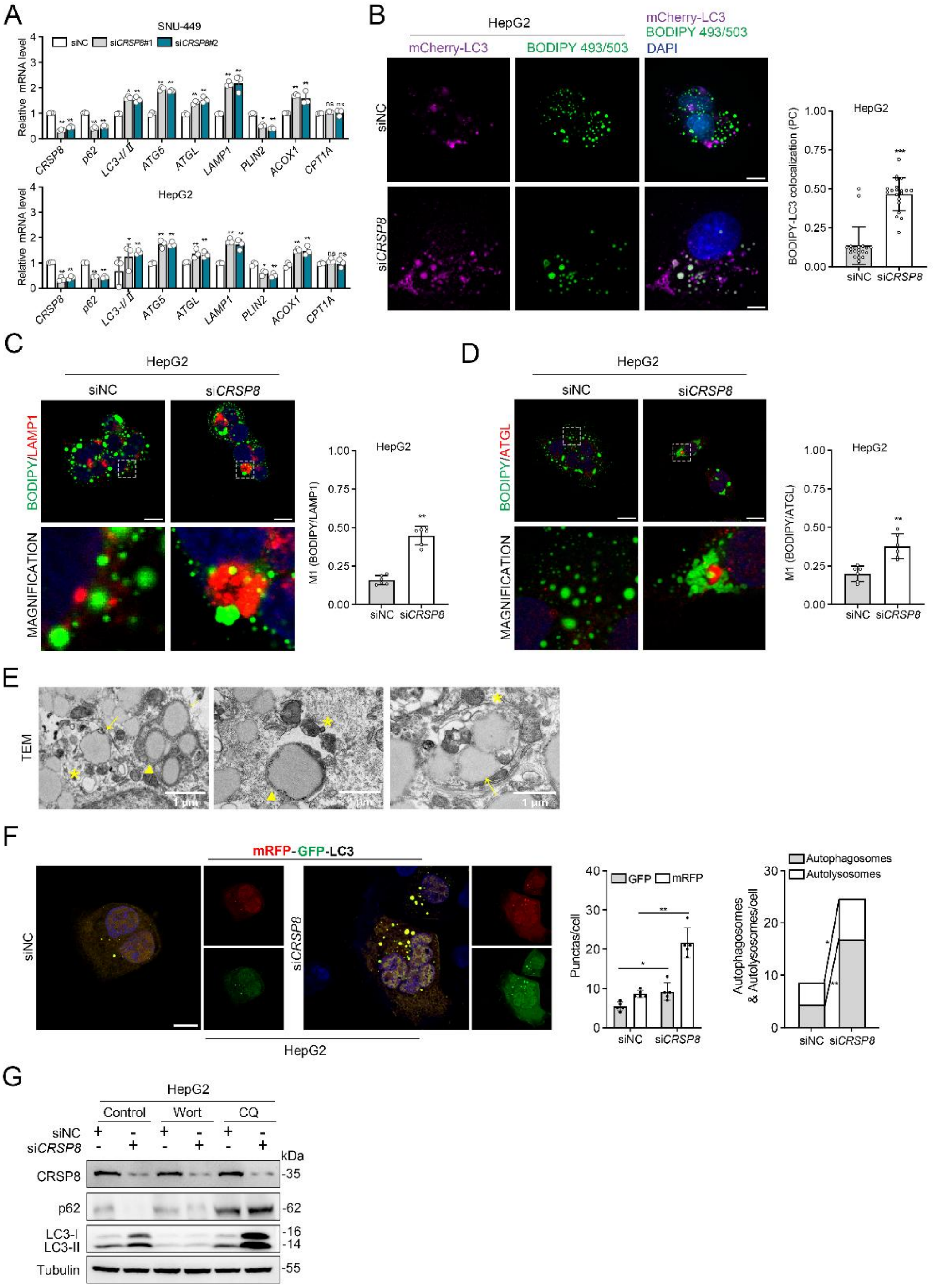

Fig. S4. ***CRSP8* knockdown activated lipophagy in HCC cells.**

(A) mRNA expressions of PPARα downstream and fatty acid oxidation related genes in SNU-449 and HepG2 cells with *CRSP8* knockdown (n = 3).

(B) Co-localization of BODIPY 493/503 staining of lipid droplets and mcherry-LC3 in the control and si*CRSP8* group of HepG2 cells (PC = Pearson coefficient). Nuclei are stained with DAPI. Scale bars represent 10μm.

(C-D) Representative co-staining of BODIPY/LAMP1 and BODIPY/ATGL in HepG2 cells. Nuclei were stained with DAPI. Scale bar, 10μm.

(E) Direct association of autophagosomes with LDs observed in electron micrographs of HepG2 cells with *CRSP8* knockdown. Arrowheads, autophagosomes; arrows, LDs; asterisks, autolysosomes. Cells were fixed with 3% glutaraldehyde with Phosphate Buffer, the ultrathin sections (70nm thick) were sectioned with microtome (Leica EM UC6) and examined by a transmission electron microscope as described in the methods with more details.

(F) Representative images and quantification of autophagic flux in HepG2 cells (n = 5 per group). Scale bar, 10μm.

(G) Western blot analysis of CRSP8, p62 and LC3 expression in the control and si*CRSP8* HepG2 cells treated with or without wortmannin (Wort) or chloroquine (CQ). Mean ± SD, n = 3 biological replicates analyzed using unpaired Student's *t*-test (two-tailed) or Mann-Whitney test (two-tailed), $*P < 0.05$; $**P < 0.01$; $***P < 0.001$.

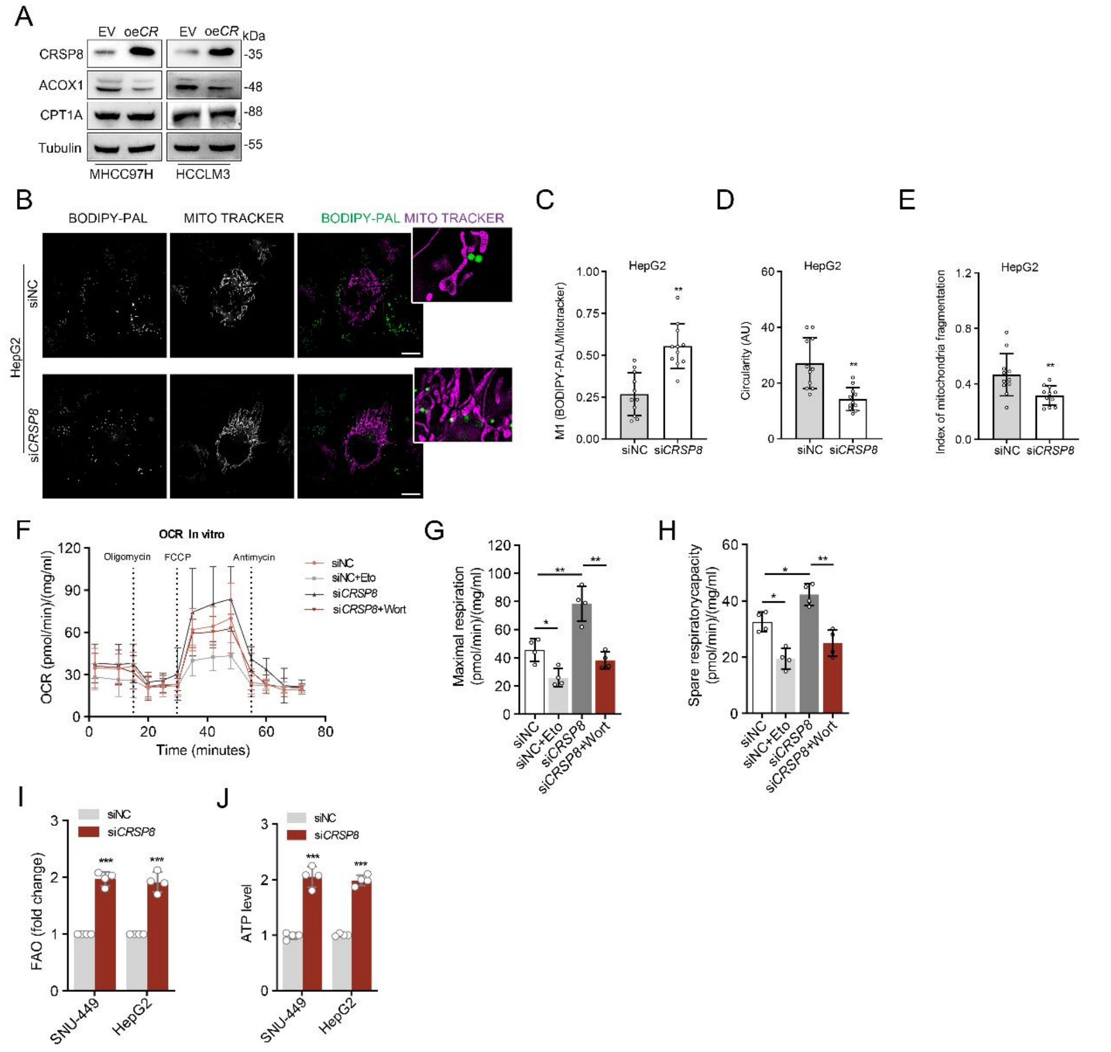

Fig. S5. ***CRSP8* knockdown activated FAO in HCC cells.**

(A) Western blotting demonstrates the effects of *CRSP8* overexpression on the expression of critical enzymes for FAO.

(B-E) Representative super resolution live images (**B**) of the control and si*CRSP8* HepG2 cells pulsed with BODIPY-PAL (overnight incubation) and imaged after 24 h chase period. Scale bar, 10μm. Mitochondria were labeled with MitoTracker deep red (30 min) before imaging, colocalization index M1 (**C**), mitochondrial circularity (**D**), and index of mitochondrial fragmentation (number of mitochondria/ total mitochondrial area) (**E**). Zoomed boxed areas show colocalization of the mitochondria (magenta) with BODIPY-PAL-signals (green). Mean ± SD, n = 3 biological replicates analyzed using unpaired student's *t*-test (two-tailed).

(F-H) Oxygen consumption rate (OCR) (**F**) of siNC or si*CRSP8* HepG2 cells measured in β-oxidation assay medium. Etomoxir (Eto, 40μM) and wortmannin (Wort) were added 15 min

prior to baseline measurement in β-oxidation assay medium. Maximal respiration (**G**) and Spare respiratory capacity (**H**) from OCR in (**F**). Mean ± SD, n = 4 biological replicates analyzed using one-way ANOVA for multiple comparisons.

(I) Fatty acid oxidation (FAO) assay of siNC or si*CRSP8* SNU-449 and HepG2 cells supplemented with 2 mCi/mL [9,10-3H]-palmitic acid for 18 h.

(J) ATP production was determined in SNU-449 and HepG2 cells transfected. Mean ± SD, n = 4 biological replicates analyzed using unpaired Student's *t*-test (two-tailed), *$P < 0.05$; *$P < 0.01$; ***$P < 0.001$.

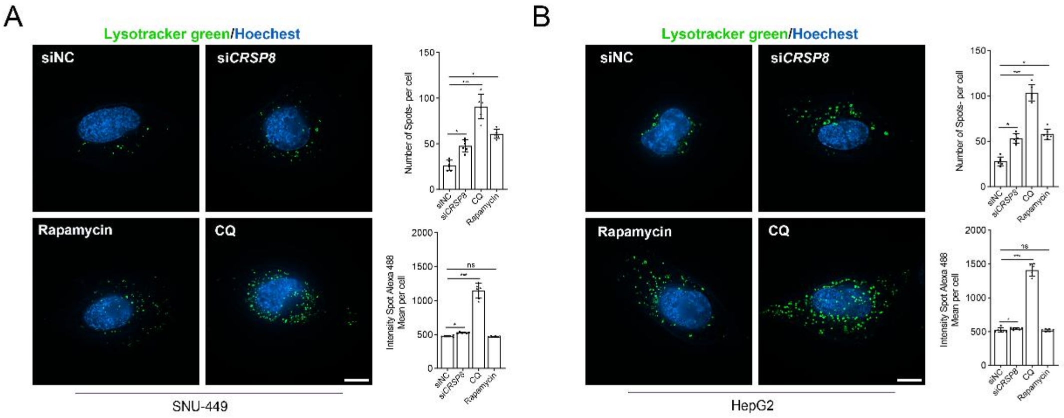

Fig. S6. The effect of *CRSP8* expression on lysosomes.

(A-B) Fluorescence microscopy images for cells stained with LysoTracker™ Green (lysosomes, green) 30 min after treatment. Right: surface plots of lysosome staining. Mean ± SD, n = 6 biological replicates analyzed using One-way ANOVA for multiple comparisons, *$P < 0.05$; **$P < 0.01$; ***$P < 0.001$.

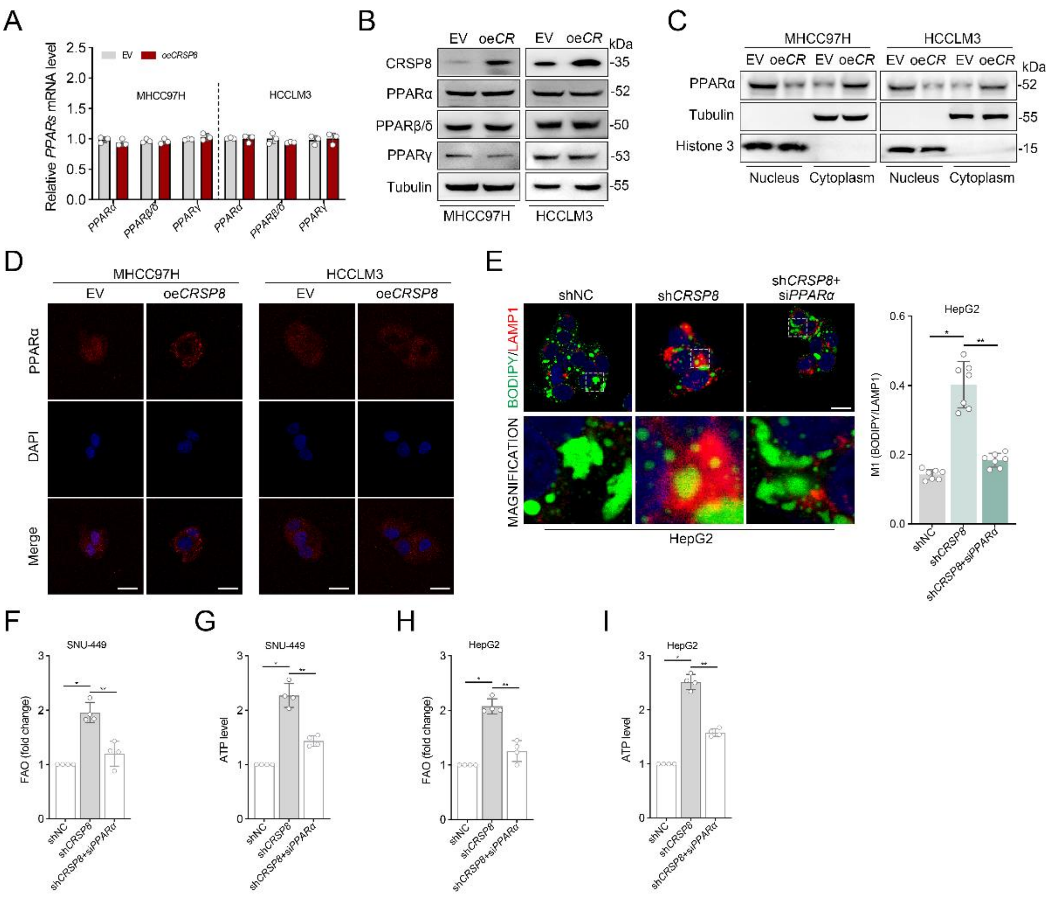

Fig. S7. Impaired lipophagy and FAO induced by *CRSP8* is hepatic nPPARα-dependent

(A-B) qRT-PCR and Western blotting verify the expression of PPARs in MHCC97H and HCCLM3 cells with *CRSP8* overexpression. Mean ± SD, n = 3 biological replicates analyzed using unpaired Student's *t*-test (two-tailed).

(C) PPARα expression in cytoplasmic and nuclear fractions, as detected by immunoblot analysis. Tubulin was used as a loading control for the cytoplasmic fraction, and Histone 3 was used as a loading control for the nuclear fraction.

(D) PPARα (red) expression in indicated MHCC97H and HCCLM3 cells, as detected by an immunofluorescence assay. The merged images show overlays of PPARα (red) and nuclear staining by DAPI (blue). Scale bar, 10μm. Mean ± SD, n = 3 biological replicates.

(E) Representative co-staining of BODIPY/LAMP1 in HepG2 cells with indicated treatment. Nuclei were stained with DAPI. Right, quantification of colocalization index M1 with ImageJ. Scale bar, 10μm. Mean ± SD, n = 7 biological replicates.

(F-I) Fatty acid oxidation (FAO) assay of SNU-449 and HepG2 cells supplemented with 2 mCi/mL [9,10-3H]-palmitic acid for 18h (**F, H**). ATP production was determined in SNU-449 and

HepG2 cells transfected (**G, I**). Mean ± SD, n = 4 biological replicates analyzed using unpaired student's *t*-test (two-tailed). *$P < 0.05$; **$P < 0.01$; ***$P < 0.001$.

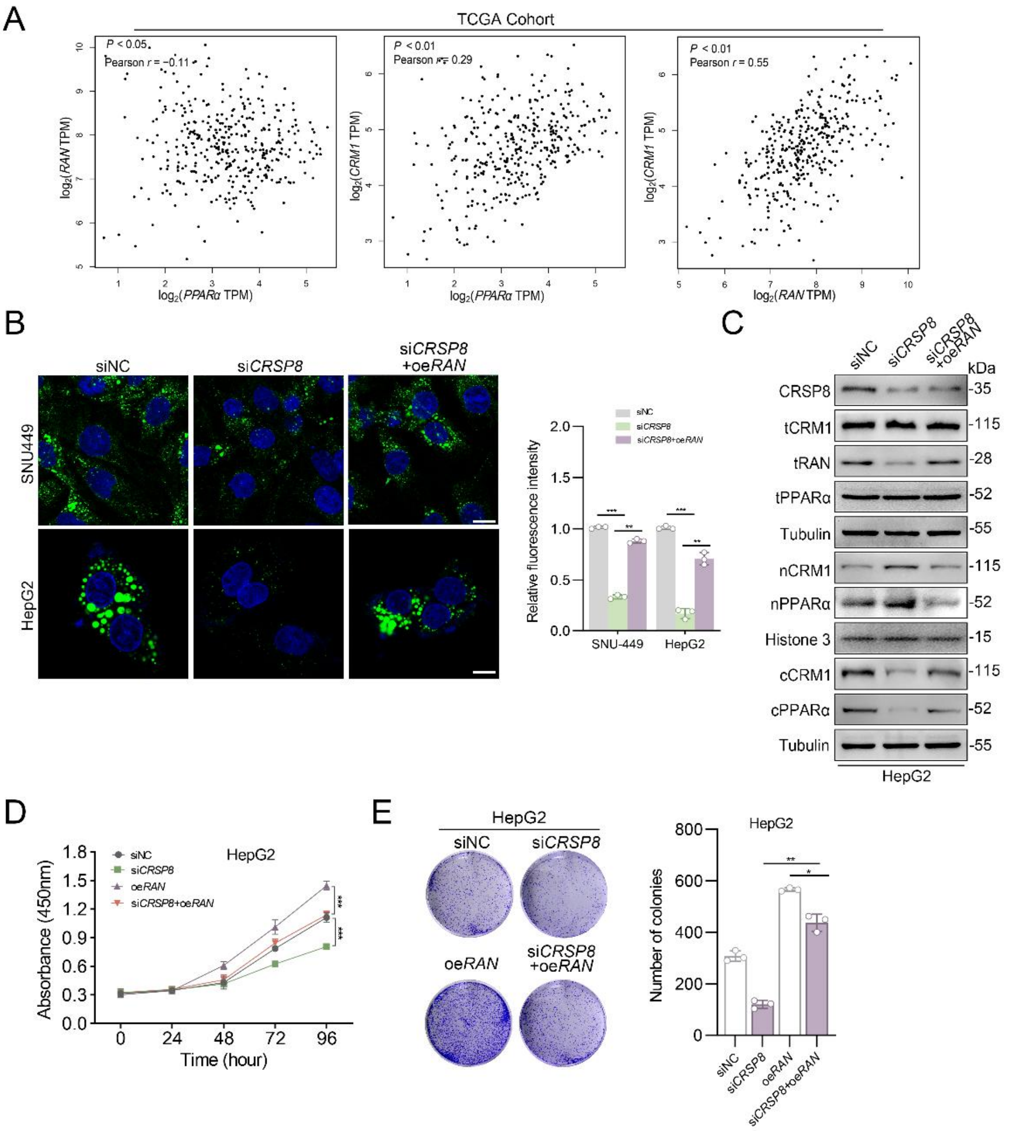

Fig. S8. ***CRSP8* regulates the nucleus-cytoplasm shuttling heterotrimer via transcriptionally activating *RAN*.**

(A) Pearson correlation analysis between the expression of PPARα, CRM1 and RAN in the TCGA-LIHC cohort.

(B) Neutral lipids stained with BODIPY 493/503 probe after *CRSP8* knockdown or *RAN* overexpression in HCC cells. Scale bar, 10μm.

(C) *CRSP*8-knockdown cells were transfected with *RAN*-overexpressed plasmid and the total, nuclear, and cytoplasmic protein levels of CRM1 and PPARα were analyzed by immunoblotting.

(D) CCK8 proliferation assays were performed in HepG2 cells with *CRSP8* knockdown or *RAN* overexpression (n = 3).

(E) Colony formation assay was performed in HepG2 cells with *CRSP8* knockdown or *RAN* overexpression. Right, quantification of colony number.

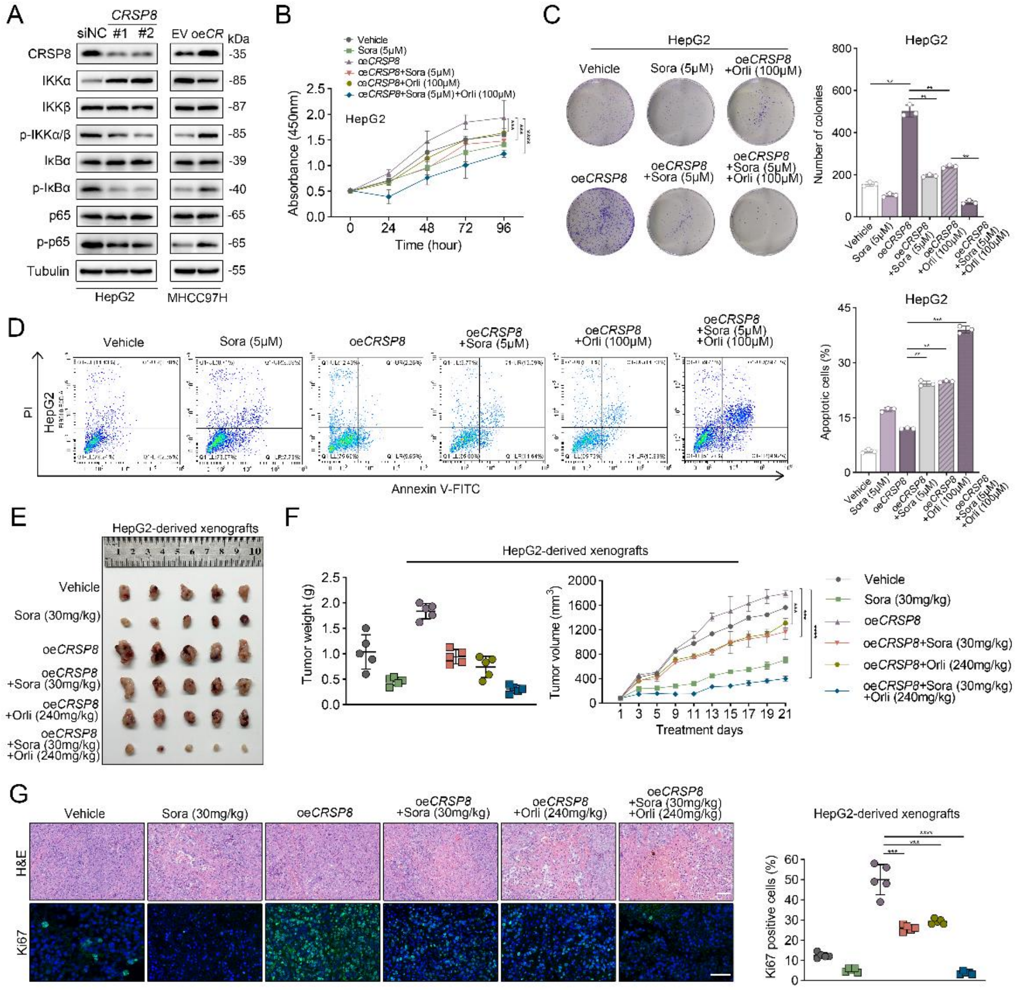

Fig. S9. **Combination of orlistat and sorafenib significantly inhibits HCC growth *in vitro* and *in vivo*.**

(A) Protein levels of phosphor-p65 and total p65 in HCC cells with CRSP8 knockdown and overexpression were analyzed by Western blot. **(B-D)** HepG2 and MHCC97H cells were treated with 100μM orlistat and 5μM sorafenib separately or in combination for indicated time, and cell proliferation was evaluated by CCK-8 assay (n = 3) **(B)** and colony formation assay **(C)**. HepG2 and MHCC97H cells were treated with 100μM orlistat and 5μM sorafenib separately or in combination for 48h, and apoptosis was analyzed by flow cytometry (n = 3) **(D)**. **(E-G)** Nude mice bearing HepG2-derived xenografts were intraperitoneally administered orlistat (240mg/kg) and/or orally administered sorafenib (30mg/kg) every day, n = 5. Representative images of the tumors **(E)**, tumor growth curves and tumor weight **(F)**, representative images of hematoxylin and eosin **(G)** (Scale bar, 100μm) and Ki67 staining **(G)** (Scale bar, 50μm) in tumor tissues are shown (n = 5). Data are analyzed using unpaired student's *t*-test (two-tailed). *$P < 0.05$; **$P < 0.01$;

***$P < 0.001$.
